# The Impact of Modern Admixture on Archaic Human Ancestry in Human Populations

**DOI:** 10.1093/gbe/evad066

**Published:** 2023-04-27

**Authors:** Kelsey E Witt, Alyssa Funk, Valeria Añorve-Garibay, Lesly Lopez Fang, Emilia Huerta-Sánchez

**Affiliations:** Ecology, Evolution, and Organismal Biology, Brown University, Providence, Rhode Island; Center for Computational Molecular Biology, Brown University, Providence, Rhode Island; Center for Computational Molecular Biology, Brown University, Providence, Rhode Island; Molecular Biology, Cell Biology, & Biochemistry, Brown University, Providence, Rhode Island; Center for Computational Molecular Biology, Brown University, Providence, Rhode Island; Licenciatura en Ciencias Genómicas, Escuela Nacional de Estudios Superiores Unidad Juriquilla, Universidad Nacional Autónoma de México, Querétaro, Mexico; Laboratorio Internacional de Investigación sobre el Genoma Humano, Universidad Nacional Autónoma de México, Querétaro, Mexico; Department of Life & Environmental Sciences, University of California, Merced, California, United States of America; Ecology, Evolution, and Organismal Biology, Brown University, Providence, Rhode Island; Center for Computational Molecular Biology, Brown University, Providence, Rhode Island

**Keywords:** admixture, archaic introgression, population genomics

## Abstract

Admixture, the genetic merging of parental populations resulting in mixed ancestry, has occurred frequently throughout the course of human history. Numerous admixture events have occurred between human populations across the world, which have shaped genetic ancestry in modern humans. For example, populations in the Americas are often mosaics of different ancestries due to recent admixture events as part of European colonization. Admixed individuals also often have introgressed DNA from Neanderthals and Denisovans that may have come from multiple ancestral populations, which may affect how archaic ancestry is distributed across an admixed genome. In this study, we analyzed admixed populations from the Americas to assess whether the proportion and location of admixed segments due to recent admixture impact an individual's archaic ancestry. We identified a positive correlation between non-African ancestry and archaic alleles, as well as a slight increase of Denisovan alleles in Indigenous American segments relative to European segments in admixed genomes. We also identify several genes as candidates for adaptive introgression, based on archaic alleles present at high frequency in admixed American populations but low frequency in East Asian populations. These results provide insights into how recent admixture events between modern humans redistributed archaic ancestry in admixed genomes.

**Significance**

Studies of how archaic ancestry is distributed across modern populations often focus on individuals without a history of recent admixture. Our study of archaic ancestry in admixed individuals in the Americas demonstrates that there is more archaic ancestry in regions of the genome derived from populations with a history of archaic gene flow (like Europeans or Indigenous Americans) compared with genome regions derived from populations with little archaic gene flow (like Africans). We also were able to focus on regions of Indigenous American ancestry to detect archaic variants that were possibly targeted by positive selection prior to European colonization.

## Introduction

Admixture, or the genetic merging of two or more parental lineages, has been demonstrated as an important contributor to modern human genetic variation (Rudan 2006; Hellenthal et al. 2014; Martin et al. 2017). Examination of human DNA sequence data from the past and present suggests that admixture has been more frequent than previously thought (Korunes and Goldberg 2021). For example, we now have evidence that archaic and modern humans interbred, and that numerous population replacement and admixture events occurred across Eurasia in the past, resulting in the transmission of genes between previously geographically separated populations (Bustamante and Henn 2010; Green et al. 2010; Reich et al. 2010). Many of the genomes of contemporary individuals in the Americas are the result of various admixture events due to European colonization and the transatlantic slave trade (Moreno-Estrada et al. 2013; Bryc et al. 2015; Ongaro et al. 2019), as well as continuous gene flow from a number of immigrant populations across Europe, Africa, and Asia (Han et al. 2017).

Many modern humans also show evidence of introgression, or the incorporation of alleles from archaic humans like Neanderthals and Denisovans (Green et al. 2010; Reich et al. 2010). Modern humans encountered both groups as they expanded out of Africa, and high-coverage genome sequencing of a Denisovan (Meyer et al. 2012) and multiple Neanderthals (Prüfer et al. 2014; Prüfer et al. 2017; Mafessoni et al. 2020) has helped characterize the archaic variation that remains in modern human genomes. Neanderthal ancestry is also found in some African populations via admixture with Eurasian populations that harbored archaic ancestry migrating back into Africa (Wall et al. 2013). Studies estimate that non-African populations have 1–4% Neanderthal admixture, with East Asian populations exhibiting more Neanderthal introgression than West Eurasian populations (Wall et al. 2013; Sankararaman et al. 2014), perhaps due to more archaic admixture events with ancestral East Asians, but other plausible scenarios have been proposed (Coll Macià et al. 2021; Witt et al. 2022). Denisovan ancestry, however, shows a more varied distribution: Oceanians have by far the most Denisovan ancestry (up to 5%) (Reich et al. 2011; Vernot et al. 2016), and while South Asians and East Asians also have some Denisovan ancestry, European populations have very little (Sankararaman et al. 2016; Witt et al. 2022). In some cases, these past introgression events likely resulted in adaptive introgression (Bustamante and Henn 2010; Green et al. 2010; Reich et al. 2010)—selection favoring archaic variants to facilitate adaptation to novel environments in modern humans (Racimo et al. 2015). Some candidate regions identified as adaptively introgressed include genes responsible for body fat distribution (Racimo et al. 2018), high-altitude adaptation (Yi et al. 2010; Huerta-Sánchez et al. 2014), skin pigmentation, and innate immunity (Racimo et al. 2017). Many of these adaptive archaic haplotypes are not found in all populations but are continent- or region-specific. We, therefore, observe regional differences in the frequency and distribution of archaic alleles, likely based on the geographic and temporal distance from the initial archaic admixture events, and on the past selective pressures and demographic events that a population was exposed to.

Until recently, the landscape of archaic ancestry in modern humans has primarily been studied in the context of Eurasian populations, and consequently much less is known about how archaic ancestry is distributed in populations in the Americas. Notably, these populations also harbor remnants of archaic ancestry (Sankararaman et al. 2014, 2016; Racimo et al. 2017) as they are the descendants of ancestral populations that interbred with archaic humans in the past. While previous studies show that Indigenous American individuals have a similar proportion of archaic admixture to East Asians (1.37% Neanderthal and 0.05% Denisovan ancestry in the Americas, compared with 1.39% Neanderthal and 0.06% Denisovan ancestry in East Asians; Sankararaman et al. 2016), less is known about how demography and natural selection have affected archaic variation in these populations. Interestingly, it was recently discovered that Peruvians exhibit the largest number of high-frequency–derived archaic alleles and the largest number of candidate loci for adaptive introgression (Racimo et al. 2017), further demonstrating the value of examining archaic ancestry in the Americas. One reason why many populations in the Americas are mostly excluded from studies characterizing the effects of archaic ancestry is that many populations are admixed (due to European colonization and the transatlantic slave trade), although recent advances in local ancestry inference methods have allowed for the estimation of admixed genomes into discrete ancestry tracts (Geza et al. 2019). These signals of admixture can also be indicative of selection (Tang et al. 2007), past demographic events (Vernot and Akey 2015; Wang et al. 2020), and even disease susceptibility (Rudan 2006; Mautz et al. 2019), demonstrating the value of analyzing admixed individuals.

Furthermore, more studies of admixed populations are needed as human populations are tending to become more admixed due to increased mobility, and we need to investigate how admixture shapes patterns of genetic variation. Recent admixture in the Americas likely impacted the frequency and distribution of archaic variants within a population, and studying these populations can provide more insights into the impact of this recent gene flow on admixture from archaic humans. In this study, we investigate how recent admixture has shaped the distribution of archaic variants in admixed populations in the Americas. We compare the admixed American populations in the 1000 Genomes Phase III data (CLM—Colombians from Medellin, Colombia, MXL—Mexican Ancestry from Los Angeles, PEL—Peruvians from Lima, Peru, and PUR—Puerto Ricans from Puerto Rico) (1000 Genomes Project Consortium et al. 2015) with the high-coverage Neanderthal and Denisovan genomes (Prüfer et al. 2017; Mafessoni et al. 2020). We examine how admixture proportions of European, African, and Indigenous American ancestry impact the distribution and amount of archaic variants in these populations. We find that recent admixture increased heterozygosity and the number of autosomal variants, and that the amount of archaic admixture is proportional to the amount of Indigenous American or European ancestry. Although European and Indigenous American tracts in these admixed genomes have approximately equal proportions of Neanderthal variants, Denisovan variants are found primarily in Indigenous American tracts. An analysis of putatively adaptively introgressed regions with high proportions of archaic ancestry in admixed populations suggests selection for genes related to multiple pathways including immunity, metabolism, and brain development, although no pathway was found to have statistically significant enrichment. This study demonstrates how recent admixture modulates the distribution of archaic variants in modern admixed populations.

## Results

### Effects of Recent Admixture on Modern Genomes

**Fig. 1.**
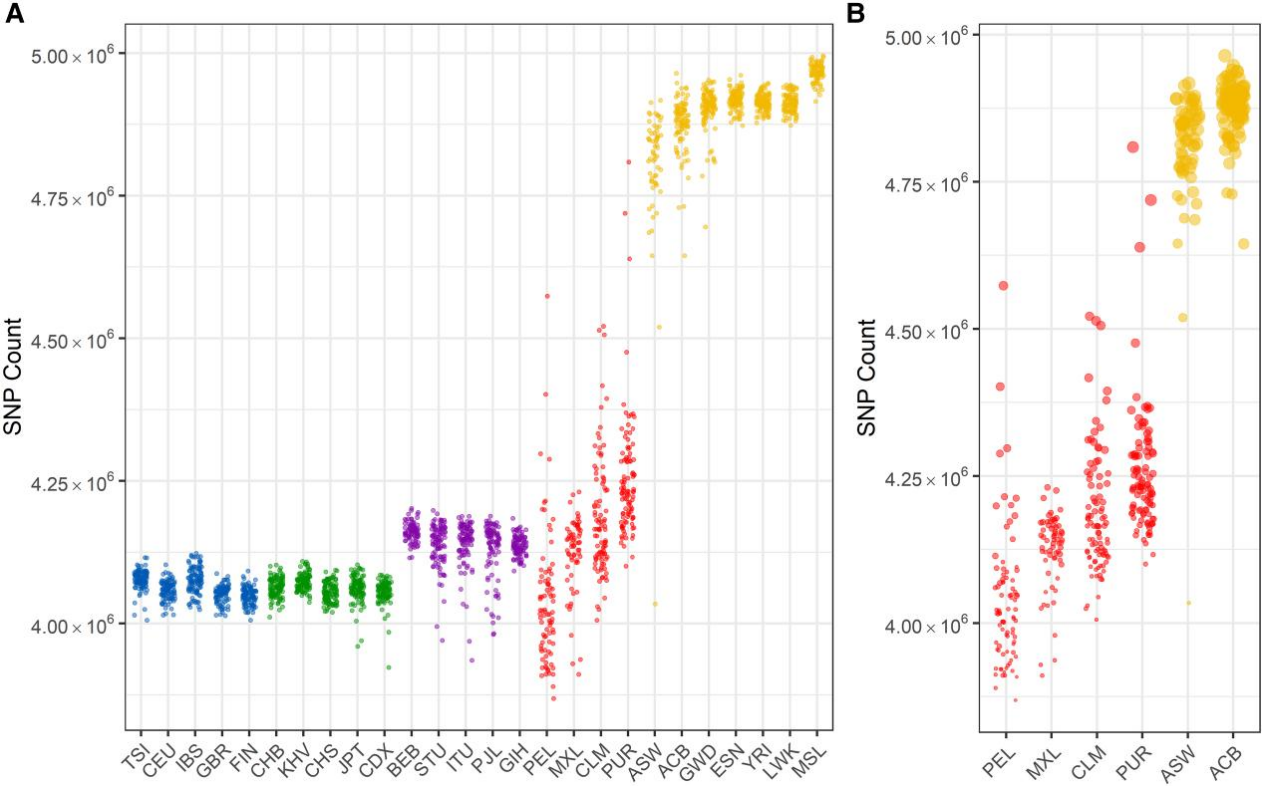
A count of sites containing at least one copy of the alternate allele for each individual. Populations are organized into superpopulations, as defined by the 1000 Genomes Project. (A) Counts for each individual in the 1000 Genomes data set, separated by population. (B) Counts for each individual in the admixed American populations, where dot size is proportional to the amount of African ancestry.

We began examining admixed populations by comparing them with the unadmixed populations sequenced by the 1000 Genomes Project. By replicating figure 1*B* from The 1000 Genomes Project Consortium 2015, we confirm that the number of autosomal variants per genome vaqries greatly across the 26 populations in the 1000 Genomes Project Phase III data set (fig. 1*A*). As expected, African individuals harbor the largest number of variants. Interestingly, individuals from the recently admixed American populations show a broader range in the total number of variants per genome across a population compared with other populations. This is likely due to the individuals within these populations having varying levels of African and Indigenous American ancestry. To explore whether the number of variants correlates with the amount of African ancestry, we examined modern ancestry tracts (African, European, and Indigenous American) that had been previously identified for the admixed 1000 Genomes individuals as defined by Martin et al. (2017) (table 1, supplementary fig. S1, Supplementary Material online), which used RFMix, a random forest-based ancestry inference method to identify haploid ancestry tracts (Maples et al. 2013). African populations and admixed populations with high African ancestry percentages (ACB, African Caribbean in Barbados; ASW, African Ancestry in Southwest US; and to a lesser extent PUR) have the greatest number of variants (fig. 1*B*). In addition to calculating the number of variants, which counts the total number of sites in the genome containing the alternate allele, we also calculated the number of heterozygous sites for each individual. Individuals of recently admixed populations from the Americas also tend to show higher proportions of heterozygous sites compared with South Asian, European, or East Asian populations (supplementary fig. S2, Supplementary Material online), likely because admixed populations can contain alleles unique to multiple geographic populations (Kidd et al. 2012). This is consistent with theoretical studies that have suggested that admixture between populations can increase heterozygosity in admixed individuals (Boca et al. 2020). Within individuals, the proportion of heterozygous sites in a genome region is also higher in regions containing African ancestry compared with regions with no African ancestry (supplementary fig. S3, Supplementary Material online).

**Table 1:** Ancestry in Admixed American Populations

		AFR	NAT	EUR	UNK
CLM	min	0.002	0.032	0.37	0
	med	0.045	0.25	**0**.**65**	0.019
	max	0.37	0.58	0.96	0.029
MXL	min	0.003	0.043	0.055	0.004
	med	0.038	**0**.**47**	**0**.**46**	0.021
	max	0.087	0.93	0.94	0.032
PEL	min	0	0.32	0.017	0
	med	0.007	**0**.**77**	0.19	0.013
	max	0.38	0.98	0.52	0.043
PUR	min	0.015	0.049	0.24	0.006
	med	0.1	0.14	**0**.**73**	0.018
	max	0.68	0.22	0.91	0.027

Note.—This table gives the minimum, median, and maximum recent ancestry for each admixed American population analyzed in this study. The values were calculated using the individual data from Martin et al. (2017). Values in bold show the largest modern ancestry component for the admixed population.

### The Effects of Admixture on Archaic Variation

**Fig. 2.**
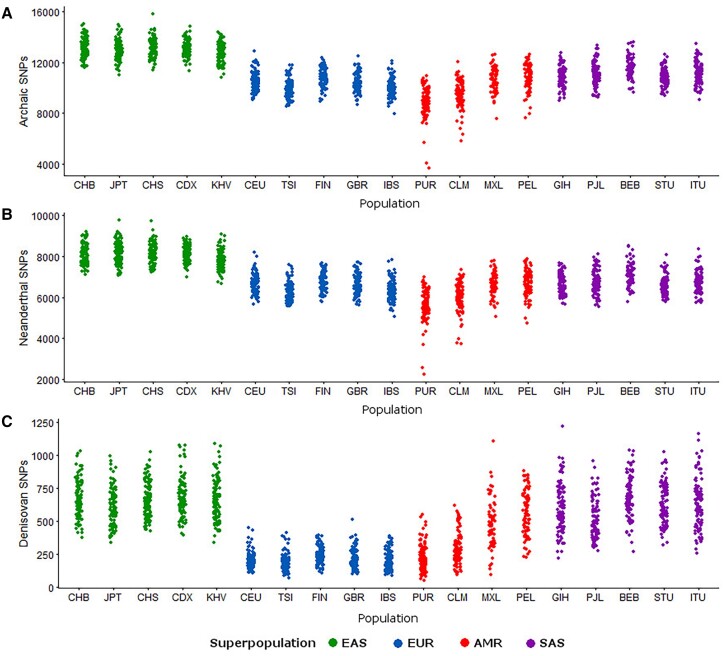
Counts of sites containing archaic alleles (Browning et al. 2018a) for all non-African individuals in the 1000 Genomes Project. Color-coding is by superpopulation, as mentioned in figure 1. (A) Counts of variants found in Neanderthals or Denisovans. (B) Counts of variants specific to Neanderthals. (C) Counts of variants specific to Denisovans.

Before looking at admixed populations specifically, we examined whether the distribution of archaic alleles varies depending on the geographic location of a population. We used the set of archaic alleles found using sPrime (Browning et al. 2018a) and calculated the number of sites with archaic alleles (all archaic, as well as Neanderthal-specific and Denisovan-specific) found in each non-African individual in the 1000 Genomes data set to examine how archaic site counts differed between populations. We found that East Asians have the greatest number of sites containing archaic alleles, followed by South Asians, while Europeans have the fewest (fig. 2*A*), as has been previously established (Sankararaman et al. 2014, 2016). The same is true for Neanderthal-specific alleles (fig. 2*B*). All populations sampled have an order of magnitude fewer Denisovan-specific variants than Neanderthal-specific variants (fig. 2*C*), and South Asians and East Asians have comparable counts of Denisovan-specific alleles, whereas Europeans have half that number. Similar to what we observe for all variants, admixed populations from the Americas have a greater range of sites containing archaic alleles per individual, whereas populations within Europe, East Asia, and South Asia show more homogeneity. For all types of archaic variants, PUR has the fewest variants, followed by CLM, MXL, and PEL, likely due to differences in the amount of Indigenous American ancestry in these populations: PEL exhibits the highest amount of Indigenous American ancestry (∼75% average) and PUR has the smallest amount (∼14%) (table 1 and supplementary fig. S1, Supplementary Material online).

**Fig. 3.**
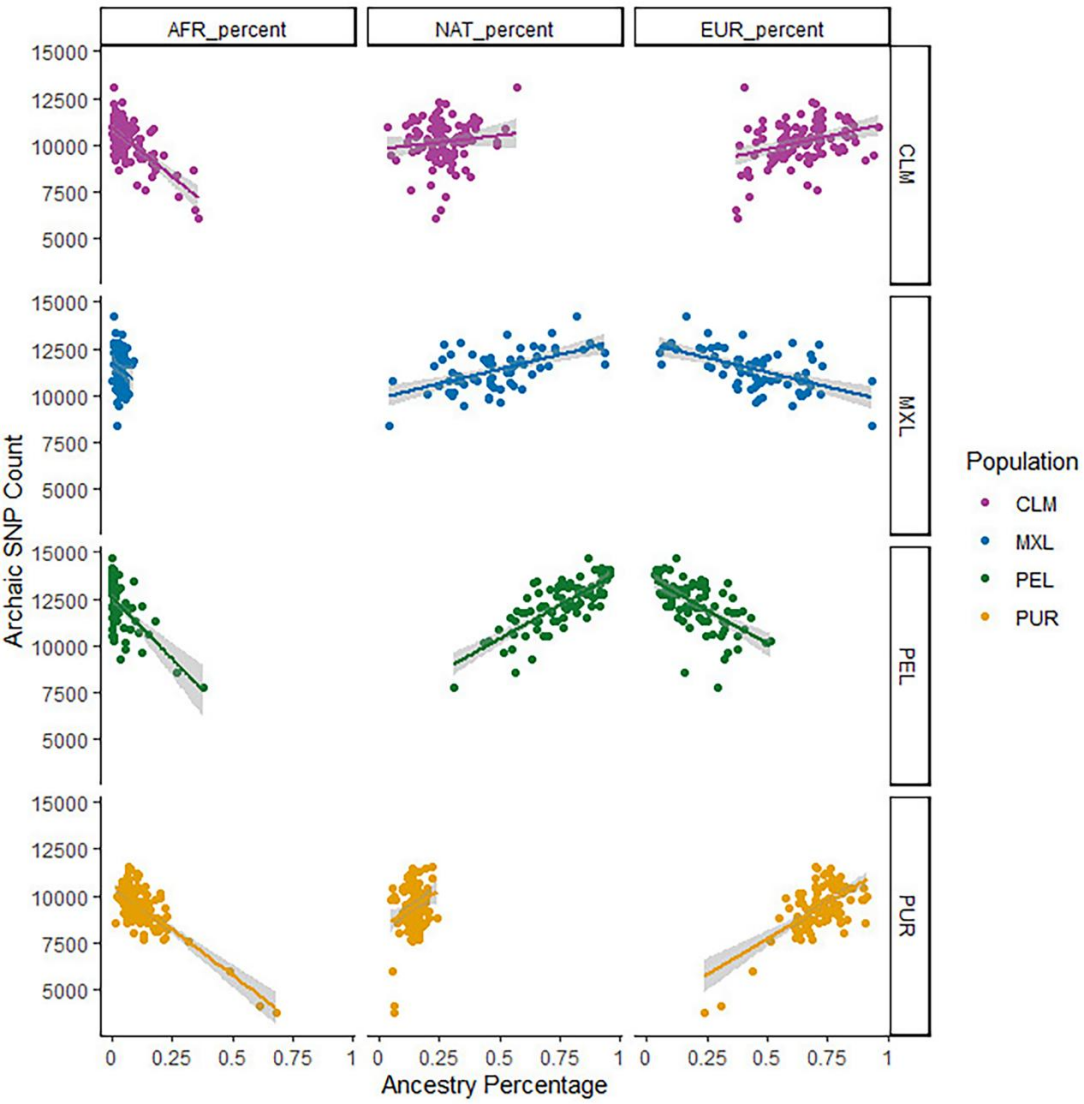
Correlations between percentages of African (AFR), European (EUR), and Indigenous American (NAT) ancestry and count of SNPs containing archaic alleles, subdivided and color-coded by population. The *x* axis is the genome-wide proportion of a given ancestry type (columns from left to right are African, Indigenous American, and European ancestry) whereas the *y* axis is the number of archaic SNPs identified for each individual using Browning et al. (2018a). Each row represents a different admixed American population (top to bottom: Colombians, Mexicans, Peruvians, and Puerto Ricans).

To explore a possible correlation between the number of Neanderthal and Denisovan alleles and the proportions of modern ancestry in admixed populations, we examined how archaic ancestry was correlated with the amount of African, European, and Indigenous American ancestry in each individual, as defined by Martin et al. (2017) (table 1 and supplementary fig. S1, Supplementary Material online). All populations show a positive correlation between Indigenous American ancestry and the total archaic allele count and a negative correlation between African ancestry and archaic alleles (fig. 3). This is expected given that Africans harbor little to no traces of Neanderthal or Denisovan introgression. Interestingly, European ancestry is positively correlated with archaic allele counts in populations with lower amounts of Indigenous American ancestry (PUR and CLM) but is negatively correlated with archaic allele counts in populations with higher proportions of Indigenous American ancestry (PEL and MXL). This is likely due to the fact that Europeans have less archaic ancestry than Indigenous Americans (Sankararaman et al. 2016), and we observe in our data that individuals with a higher proportion of Indigenous American ancestry have more archaic variation compared with those with less Indigenous American ancestry and more European ancestry (supplementary fig. S4, Supplementary Material online). For individuals with only a low percentage of Indigenous American ancestry and a high percentage of European ancestry (e.g., PUR and CLM), however, the difference in archaic ancestry proportion between Indigenous American and European ancestry segments has a negligible effect on the total archaic ancestry in each individual.

**Fig. 4.**
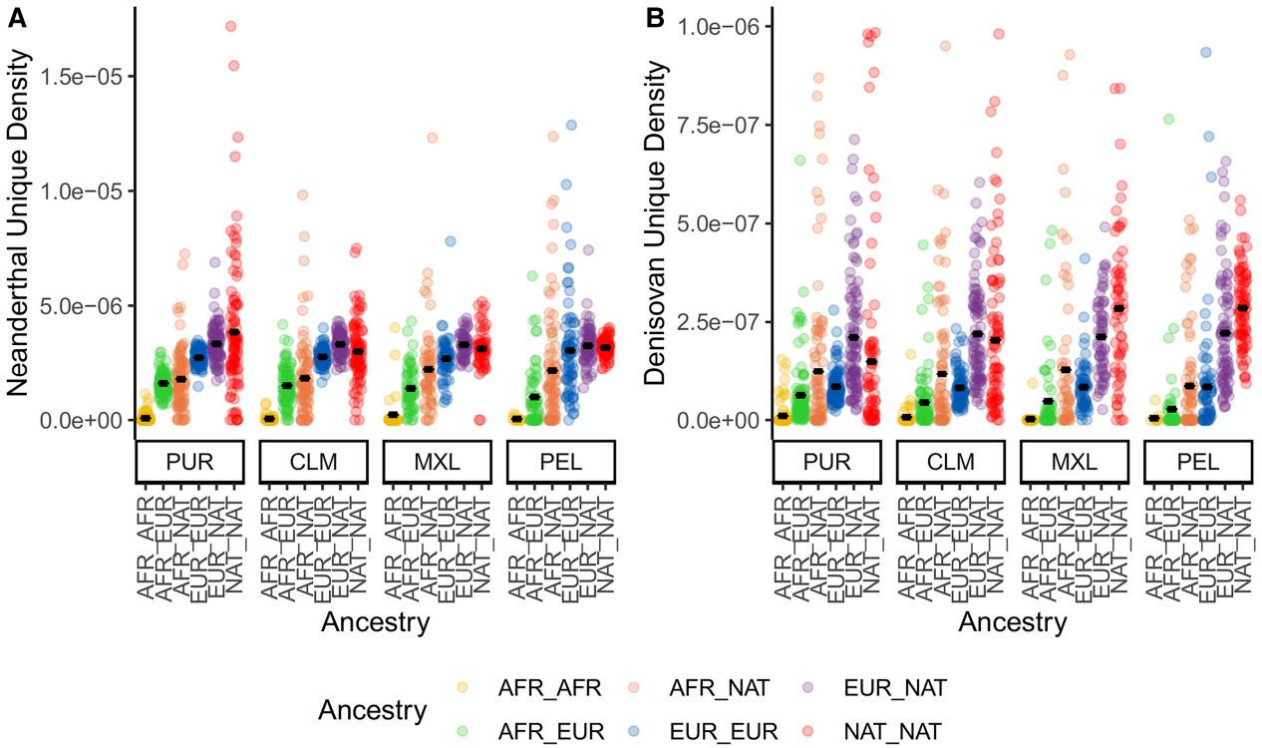
The average archaic allele density across all tracts of a given ancestry type for each individual, subdivided by population. Ancestry abbreviations are “AFR” for African, “EUR” for European, and “NAT” for Indigenous American. Archaic allele density is calculated by subdividing the tracts for each individual into the different diploid ancestry calls (e.g., African–African or African–European), summing the total number of positions containing archaic alleles across all tracts of a given ancestry type, and dividing that total by the total length of all ancestry tracts of a given ancestry type. Individual points show the archaic allele density for a given individual and ancestry source, and the overlaid line indicates the mean. (A) Neanderthal allele density. (B) Denisovan allele density.

We also assessed whether archaic variants were more likely to be present in specific regions of ancestry across individuals by counting the number of archaic variants in genome regions deriving from the different ancestries (African, European, and Indigenous American, Martin et al. 2017). The 1000 Genomes data were rephased in the process of generating the ancestry calls, and so we could not assign a specific ancestry call to each chromosome in the phased 1000 Genomes data. Instead, we determined the diploid ancestry calls for each region of the genome and used that to compare the amount of archaic variation in a given genome region with the ancestry calls in that region. We calculated “archaic allele density” in these regions by summing the number of positions with archaic alleles identified in all regions with a given ancestry designation (i.e., EUR–EUR for both chromosomes having European ancestry or AFR–EUR for one chromosome with African ancestry and one with European ancestry) and dividing it by the combined length of all regions with that ancestry designation. Consistent with the correlations identified in figure 3, regions of African ancestry are much lower in archaic allele density than regions of European or Indigenous American ancestry (fig. 4), with little to no archaic alleles present in African regions. Variance in archaic allele density is higher in populations with lower proportions of a given type of ancestry (e.g., Indigenous American ancestry in PUR or European ancestry in PEL), where “genome-wide” estimates are likely calculated with only a small number of tracts. Neanderthal-exclusive variants have an average density that is five times higher than that of Denisovans, and Neanderthal allele density is approximately equal between European and Indigenous American segments of the genome. Denisovan allele density, however, is slightly higher in Indigenous American segments than in European segments in populations with higher Indigenous American ancestry (PEL and MXL). This pattern is also observed when looking at individual ancestry tracts, rather than the density across all of an individual's tracts of a given ancestry designation—of the tracts in the top 1% for highest density (supplementary fig. S5, Supplementary Material online), only one African tract is present (which is similar to a Denisovan haplotype found in some Indigenous American individuals but likely derives from standing ancestral variation; supplementary fig. S6, Supplementary Material online), and the Indigenous American tracts with high Denisovan allele density outnumber the European tracts with high Denisovan density. A single individual had a European ancestry tract with a high proportion of Denisovan ancestry, but the haplotype in question was shared among multiple human populations, including YRI, GBR, and CHB, and had a large number of ancestral alleles, suggesting that this tract represents shared ancestral variation rather than Denisovan introgression (supplementary fig. S7, Supplementary Material online). Mean archaic allele density for a given ancestry designation (which is the total number of archaic sites in regions with that ancestry divided by the total length of all regions with that ancestry) is approximately equal across all four admixed populations (fig. 4), with the exception of Denisovan allele density, which is higher in PEL and MXL than in CLM or PUR.

We also examined the Indigenous American ancestry segments specifically for regions that had a high density of archaic alleles and were shared across multiple individuals, to identify candidate regions of archaic introgression that are present at high frequency. We identified a total of five genomic regions that had high archaic allele density in regions of Indigenous American ancestry and were shared between at least 20 admixed individuals: three for Neanderthal alleles and two for Denisovan alleles (table 2). Of these five regions, two contain smaller segments that have been previously targeted as candidate regions for adaptive introgression (Racimo et al. 2017).

**Table 2:** A List of the Genome Regions with High Archaic Allele Density That Are Found in at Least 20 Individuals from PEL, MXL, CLM, and PUR in Indigenous American Ancestry Tracts

Archaic Source	Chromosome	Site Range	Genes
Neanderthal	2	238,150,000–243,000,000	COL6A3, MLPH, RAB17, UBE2F, SCLY, ESPNL, KLHL30, FAM132B, ILKAP, HES6, PER2, TRAF3IP1, ASB1, TWIST2, HDAC4, NDUFA10, OR6B2, OR6B3, MYEOV2, OTOS, GPC1, ANKMY1, DUSP28, RNPEPL1, CAPN10, GPR35, AQP12B, KIF1A, AGXT, **PASK**, **SNED1**, **MTERFD2**, **PPP1R7**, **ANO7**, **HDLBP**, **SEPT2**, **FARP2**, **STK25**, THAP4, ATG4B, DTYMK, D2HGDH, GAL3ST2, ING5, PDCD1, and NEU4
	3	194,650,000–195,550,000	XXYLT1, ACAP2, PPP1R2, APOD, SDHAP2, MUC20, and MUC4
	18	76,450,000–78,000,000	SALL3, ATP9B, NFATC1, CTDP1, KCNG2, PQLC1, HSBP1L1, TXNL4A, RBFA, ADNP2, and PARD6G
Denisovan	7	147,200,000–147,800,000	CNTNAP2
	12	37,850,000–41,650,000	ALG10B, CPNE8, KIF21A, ABCD2, SLC2A13, **LRRK2**, **MUC19**, CNTN1, and PDZRN4

Note.—The coordinates correspond to the hg19 genome build. Gene names in bold have been implicated as targets for adaptive introgression by other studies (Sankararaman et al. 2016; Racimo et al. 2017). Archaic allele density was calculated for 50-kB windows across the genome, so these genome regions represent multiple consecutive 50-kB windows that had high archaic allele density in Indigenous American ancestry tracts for at least 20 individuals.

### Identifying Local Adaptation with Archaic Alleles

We wanted to assess the possibility of adaptively archaically introgressed regions that were locally adaptive to environments in the Americas. If there are loci adaptive specifically for American populations, we would expect them to show signs of positive selection and have archaic allele frequencies in American populations that are higher than the allele frequencies in closely related populations outside of the Americas. Previous work by Racimo et al. (2017) identified a number of 40-kb putative introgressed regions in the 1000 Genomes populations. These candidate regions were identified as having the top 0.1% of number (and frequency) of shared alleles between the test and archaic population that are absent in African populations, and PEL had the largest number of candidate regions identified (Racimo et al. 2017). We chose to use the population branch statistic (PBS) to scan these regions for archaic alleles that show a signal of positive selection. This method requires the use of three populations: the population of interest, a closely related population for comparison, and an outgroup population. We used PEL and MXL as our Indigenous American populations, as those two populations have the highest average proportion of Indigenous American ancestry of all the admixed populations (table 1), and used CEU as our outgroup population. Although modern-day Siberians are the closest relatives to modern Indigenous Americans (Malyarchuk et al. 2011; Sikora et al. 2019), there is a limited number of Siberian genomes that can be used as a comparative population (Mallick et al. 2016). Therefore, we use an East Asian population (CHB) as a proxy for the ancestral population to the founding American populations, which diverged from the Siberian population ancestral to Indigenous Americans ∼30,000 years before present (Moreno-Mayar et al. 2018; Sikora et al. 2019). We also calculated the archaic allele frequency difference between the American population and CHB to ensure that sites that appear to be positively selected actually have a higher archaic allele frequency in the American population. For comparison, we additionally calculated the allele frequency difference for these populations for alleles that are rare in Africa (*f* < 0.01) but not shared with archaic genomes, to assess whether archaic and nonarchaic alleles as a whole have experienced different selective pressures (supplementary fig. S9, Supplementary Material online). New variants that have arisen in populations since humans expanded out of Africa are due either to novel mutation or gene flow from archaic humans, and so we would expect both of these variants to be impacted by the same demographic events, such as bottlenecks. A lower allele frequency difference in archaic variants would suggest that the archaic variants had a more negative impact on fitness.

**Fig. 5.**
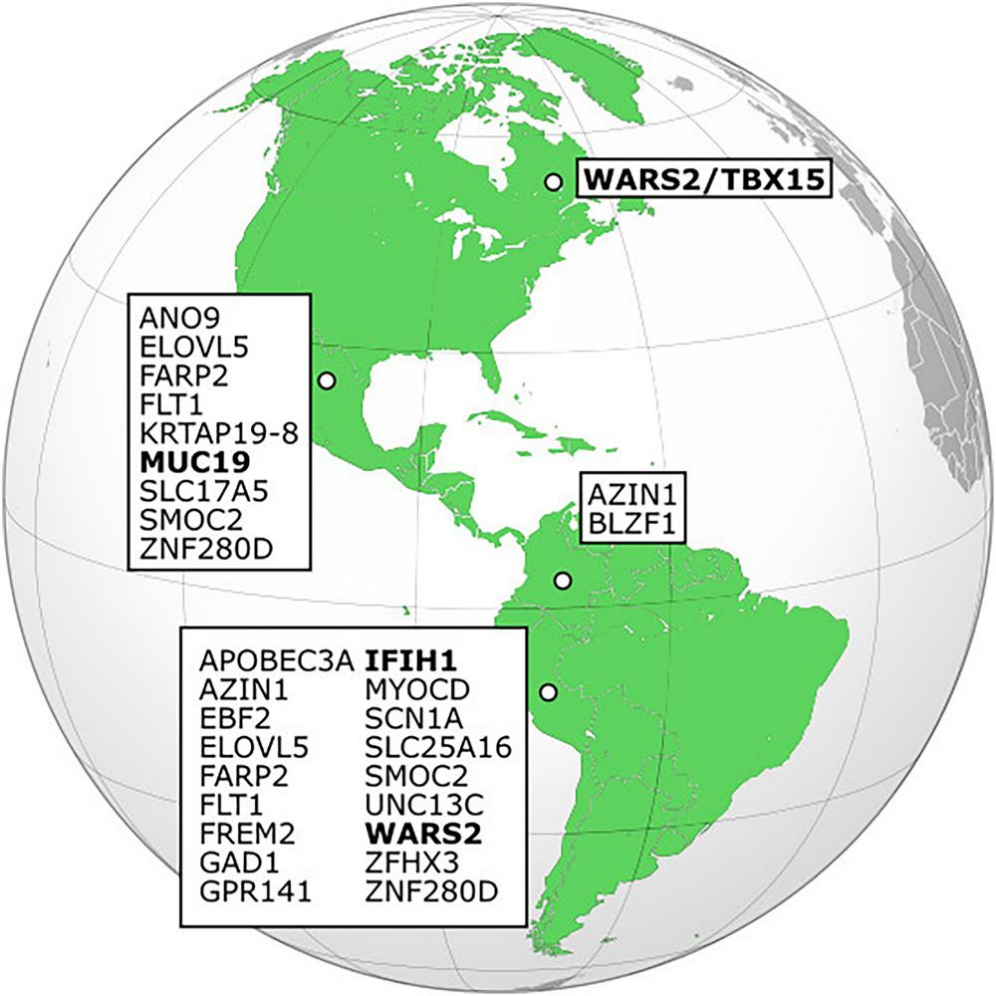
A map depicting the genes identified as adaptively introgressed in American populations. Any genes highlighted in bold have also been identified in other studies. The genes identified in this study are part of larger regions that were significant, so other nearby genes may also/instead be adaptively introgressed.

We were able to identify a number of archaic alleles that have a significantly higher allele frequency in the American populations compared with CHB, which suggests they may have been positively selected (supplementary table S1, Supplementary Material online). Some of these regions contain genes that have already been identified as adaptively introgressed in Indigenous American populations, including *IFIH1/FAP* (Ávila-Arcos et al. 2020) and *WARS2* (Racimo et al. 2018), identified in PEL, and *LRRK2/MUC19* (Reynolds et al. 2019), identified in MXL. Other genes that were identified as candidates for adaptive introgression include a region identified in PEL and MXL that contains *FARP2* (regulates cytoskeletal formation), a region in PEL and MXL that contains *PAX3* (a transcription factor important to development), a region in PEL and MXL that contains *CNTNAP2* (which affects cell receptors in the nervous system and has been implicated in neurodevelopmental disorders), and a region in PEL that contains *MYOCD*, a gene involved in cardiac function (fig. 5). A Gene Ontology (GO) enrichment analysis yielded no pathways that were significantly overrepresented, and annotation of the archaic single nucleotide polymorphisms (SNPs) within these statistically significant genes showed that 80% of the SNPs were either intronic or intergenic, whereas only 4% were found in exons (supplementary fig. S8, Supplementary Material online). Of those 80 exonic SNPs, we identified one loss of a start codon in *OXCT1* (rs4957429), which is involved in ketone metabolism, and five missense mutations. Three of the mutations (rs3750904, rs4369876, and rs12478318) were found in *SCN9A*, a sodium channel involved in the perception of noxious stimuli, one (rs61742833) was located in *PTDSS2*, which is involved in cell signaling, and one (rs2114566) was located within *MUC19*. The distributions of allele frequency differences between American populations and CHB are fairly similar across archaic and nonarchaic alleles, and they have similar mean and standard deviation values (supplementary fig. S9, Supplementary Material online). We also checked to see which genes remained significant using Mexicans as the target population and Siberians as a comparative population for the PBS analysis and confirmed significant results for multiple genomic regions that were consistent with our PBS analysis using CHB while also identifying a few additional genes that were not found in the original analysis (supplementary fig. S10 and table S2, Supplementary Material online). The regions we identify contain many genes, however, and so other genes may also (or instead) be the source of the introgression signal we observe.

The pattern of multiple alleles found at high frequency in a given genomic region that we observed can also be produced by heterosis, where recessive deleterious variants present in both a donor and a recipient population are masked as a result of admixture. When admixture occurs, the higher heterozygosity can mask the deleterious variation, showing a similar pattern to positive selection, especially in regions with high exon density and low recombination rate (Zhang et al. 2020). As heterosis is most likely to occur when recombination rates are low and the exon density in the region is high, we examined the recombination rate and exon density of the possible targets of adaptive introgression (i.e., candidate regions exhibiting high-frequency archaic alleles). We found that only two of the SNPs (∼2.4%) were in genomic regions above the 90th percentile for high exon density and low recombination rate (recombination rates of 8.22 × 10^−9^ for *CYP2B6* and 9.16 × 10^−9^ for *SPRR2F*). This suggests that, given the features of the genome region they are in, the majority of these candidate regions cannot be explained by heterosis.

**Fig. 6.**
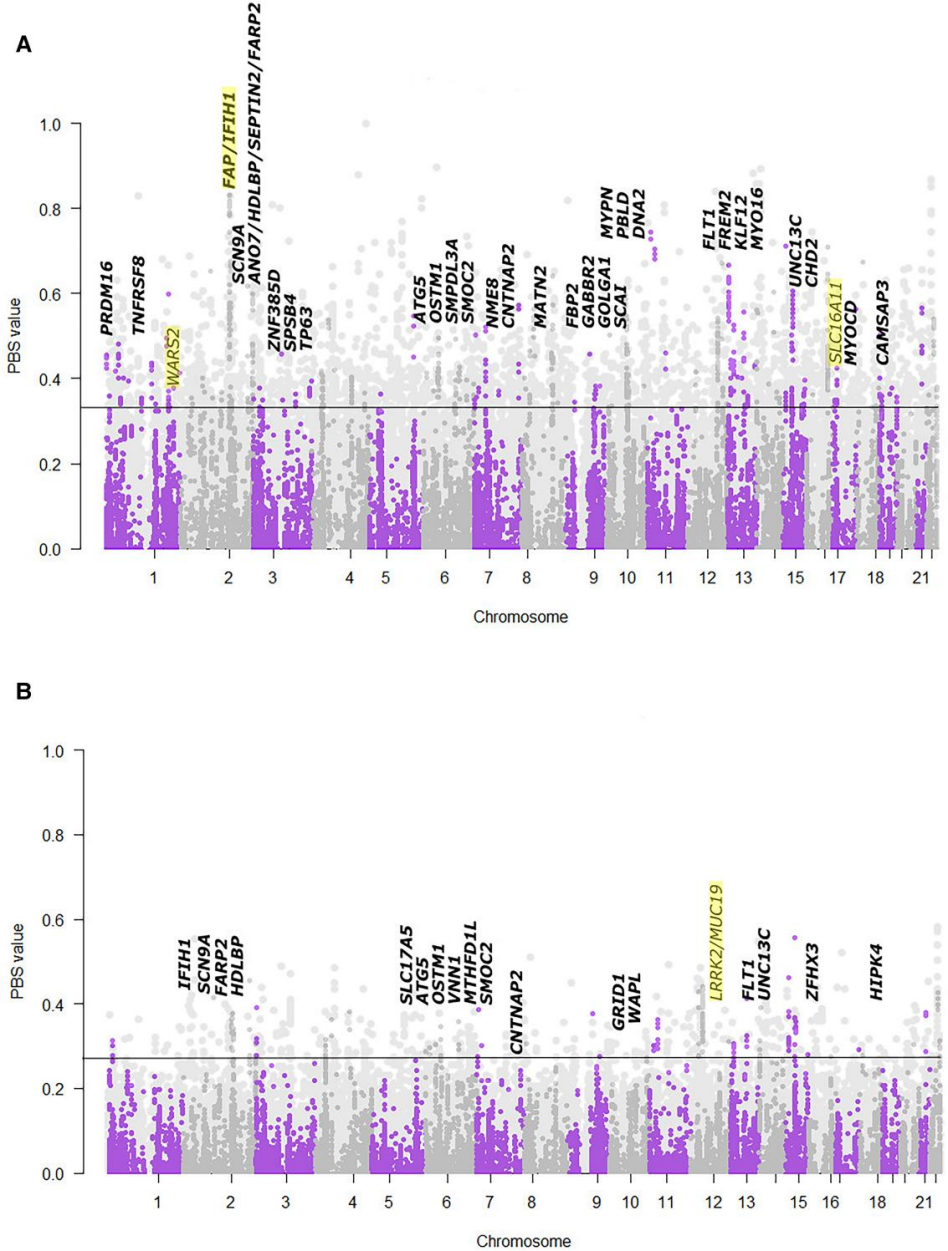
PBS results comparing the American population (A: PEL, B: MXL) with CHB (and CEU as outgroup). Bold labels are genes with at least ten archaic alleles within a gene that have PBS values above the 95th percentile of randomly selected sites that are rare in African populations and greater than 1% frequency in non-African populations. Italicized (nonbold) gene names have ten archaic alleles within 100 kb of a gene but not in the gene itself. Genes highlighted in yellow have been previously identified as putatively under selection. Lighter background dots show PBS values calculated for all segregating sites across the population's genome, and the horizontal line shows the threshold for the top 5% of all PBS values calculated at each variant site in genomes for the population. Chromosomes are illustrated by alternating colors.

It is also possible that these alleles are actually neutral and present at a higher frequency due to demographic effects, such as a strong bottleneck. To control for this, we used neutral simulations to model the longest and shortest tract that we identified using PBS statistics that contains a gene. We modeled MXL because it has previously been used to infer admixture parameters between Africans, Europeans, and Indigenous Americans (supplementary table S3 and fig. S11, Supplementary Material online) and simulated neutral alleles across the tracts and calculated PBS values for the alleles. The 95th percentile PBS values varied from 0.078 to 0.082 using a sex-averaged recombination map of the region and from 0.0.083 to 0.088 using a uniform recombination rate (supplementary fig. S12, Supplementary Material online), which are lower cutoffs than the one we observed using a set of random alleles (fig. 6).

### Comparison with Archaic Ancestry Tract Results

While the study of archaic SNPs provide valuable insights into archaic variation in modern humans, it is possible that some of these SNPs do not actually represent archaic introgression. A SNP that is rare in Africans but shared between archaic and modern humans may represent incomplete lineage sorting, meaning that the SNP was found in the ancestor of modern and archaic humans but lost in the African lineage. Therefore, we used archaic introgression tracts identified by two different studies (Sankararaman et al. 2014; Skov et al. 2018) to further interrogate our results. We first assessed how the overlap between modern ancestry segments and archaic ancestry segments was impacted by the source of modern ancestry and found that regions without African ancestry were more likely to overlap with an archaic ancestry tract, although archaic ancestry tracts could also be found in regions with African ancestry (supplementary fig. S13 and table S3, Supplementary Material online). These tracts may represent incorrectly-identified tracts of archaic ancestry, regions of the genome with ancestry that is not actually African in origin, or cases where the region is introgressed but also contains variants that are also still found in Africans. Denisovan ancestry tracts were also much more likely to be contained in regions of European or Indigenous American ancestry and were slightly less common in regions of European ancestry (supplementary fig. S14, Supplementary Material online). We also compared the archaic alleles with high PBS values with the archaic ancestry tracts identified by Skov et al. (2018) and found that 79% of the sites identified in PEL and 81% of the sites identified in MXL also overlapped with archaic ancestry tracts.

## Discussion

The increased variant count in African populations is consistent with the out-of-Africa model of human migration across the world, as well as the decreased genetic diversity in non-African populations as a result of the bottlenecks that occurred as humans moved into Eurasia, Oceania, and the Americas (Ramachandran et al. 2005; DeGiorgio et al. 2009; Li and Durbin 2011). Admixed individuals in the Americas show the second highest level of autosomal variants, consistent with their proportions of African ancestry. The admixed populations also show higher variance in the number of segregating sites per individual (figs. 1 and 2), as each individual in the population has varying contributions of African, European, and American ancestries.

The amount of archaic variation present in an admixed individual is also impacted by their proportions of African, European, and Indigenous American ancestry. As expected, African ancestry is negatively correlated with the number of archaic variants, and regions of the genome identified as African have few archaic variants, while non-African ancestry is positively correlated with archaic variation. The increased Denisovan variation in Indigenous American ancestry tracts compared with European ancestry tracts suggests that Indigenous Americans have similar archaic allele frequencies to other Asian populations, consistent with their shared history (Dulik et al. 2012; Raghavan et al. 2014). Two populations, PEL and MXL, show a slight negative correlation between European ancestry and Neanderthal variants, suggesting that Indigenous American tracts are contributing more to archaic ancestry than European tracts in populations with high Indigenous American ancestry.

We find that Denisovan allele density is lower than Neanderthal allele density in the ancestry tracts we examined (fig. 4). Part of this lower density can likely be attributed to a lower proportion of Denisovan ancestry compared with Neanderthal ancestry for European and Indigenous American individuals (Sankararaman et al. 2016). However, it has been previously demonstrated that the sequenced Denisovan genome is more divergent from the Denisovan segments in modern humans than the divergence between the sequenced Neanderthal genomes and Neanderthal segments in modern humans, and so that likely also impacts our identification of Denisovan alleles (Browning et al. 2018a, 2018b). When we used archaic introgression tracts instead of SNPs to compare modern and archaic ancestry, we found that archaic tracts were found more commonly in regions with European or Indigenous American ancestry (supplementary fig. S13 and table S4, Supplementary Material online). However, archaic introgression tracts overlapped with regions of African ancestry at only a slightly lower rate than they overlapped with other modern ancestry tracts, suggesting that some modern ancestry regions are misidentified as African or that some archaic introgression tracts are incorrectly identified as introgressed. We also found that Denisovan ancestry was more often contained in regions of Indigenous American ancestry than European ancestry and rarely found in African ancestry regions (supplementary fig. S14, Supplementary Material online).

Our analysis of the distribution of allele frequency differences between admixed American populations and East Asians showed that archaic alleles had a comparable allele frequency difference compared with alleles that were rare in Africa but not archaic in origin (supplementary fig. S9, Supplementary Material online). However, a small proportion of SNPs with archaic alleles were at much higher frequency in American populations than Han Chinese individuals, suggesting that positive selection may also have occurred for some alleles. Many of these alleles were over 2 standard deviations from the mean of the allele frequency differences. We used PBS to identify possible candidates for adaptive introgression that were unique to admixed American populations and identified alleles in multiple genes that have already been identified as targets of adaptive introgression (Racimo et al. 2017, 2018; Ávila-Arcos et al. 2020), as well as in novel candidate genes, including some that govern development and cardiac function. Some of these genes, including *FARP2*, *MUC19*, and *CNTNAP2*, were also found in Indigenous American ancestry regions with a high proportion of archaic variants (table 1). We do want to note that each of these genes was identified as part of larger regions with multiple genes, and that other genes could also be contributing to the signal of positive selection that we are observing (table 2). The fact that these alleles are found at a high frequency in admixed American populations and that they are specifically found in Indigenous American ancestry segments supports the hypothesis that archaic alleles in these regions may have been adaptive specifically for Indigenous American populations. Some of the genes we identified remained significant when we focused on allele frequency differences between Siberians and Indigenous Americans, suggesting that some alleles may have been under selection in Siberian populations as well (supplementary fig. S10 and table S2, Supplementary Material online). Still others were significant only in the comparison between Mexicans and Siberians, which may indicate that they were only selected for after humans had entered the Americas.

This study of archaic ancestry in admixed populations illustrates how a recent history of admixture can impact variation introduced from archaic humans. First, by partitioning the admixed genomes into regions corresponding to different ancestries, we find that each modern ancestry component within an admixed population mirrors what we see in the unadmixed ancestral population. For example, archaic ancestry is found almost exclusively in non-African populations and is nearly absent from genomic regions with African ancestry in admixed individuals as well. This suggests that admixed individuals can be informative about the amount of archaic ancestry in multiple ancestral populations.

However, although we were able to observe the archaic ancestry present in the separate ancestry components of an admixed individual's genome, they are imperfect representations of the populations the ancestry sources represent. For example, the Indigenous American ancestry segments found in modern individuals likely represent only a fraction of the genetic diversity found in Indigenous Americans prior to European colonization. We would therefore expect precolonization Indigenous American populations to have even more archaic variants, especially because Indigenous American ancestry is positively correlated with archaic ancestry in admixed American populations (fig. 3). Also, by examining the archaic ancestry present in ancient Indigenous Americans, we can perhaps identify additional introgressed regions that were targets of positive selection prior to European colonization. We also acknowledge that in this study, we focus on populations that all have the same admixture sources and timing, and other admixed populations with a different demographic history (such as ancestry sources that are more closely related) may not show the same clear differences in archaic ancestry distribution across different modern human ancestry tracts. Future work that examines the changes in archaic ancestry through time in admixed populations, or work that examines populations other than admixed Americans, will help clarify how recent admixture in modern humans affects the amount and distribution of archaic ancestry in the genome.

In summary, we have shown how recent admixture events impacted archaic ancestry in admixed individuals. We find that the proportion of African to non-African ancestry is proportional to heterozygosity and the number of variants and inversely proportional to the amount of archaic ancestry. We also identify a number of candidate loci that may have been adaptively introgressed, and further exploration of these variants will contribute to a better understanding of the evolutionary processes (such as the timing of admixture and strength of selection) (Corbett-Detig and Nielsen 2017; Zhang et al. 2021) and whether these variants contribute to complex traits in modern humans. Many individuals living today have ancestries deriving from multiple populations, and additional studies to characterize the impacts of admixture on an individual's genetic variation are needed to gain a better understanding of modern human diversity and its role in disease risk.

## Materials and Methods

### Characterizing Genetic Diversity in 1000 Genomes Populations

In this study, we focus on biallelic sites at SNPs, so any reference to an “allele” would refer to the variant at a single nucleotide position. We aimed to better understand patterns of genetic variation present in the 1000 Genomes Project Phase III data set by counting the number of autosomal biallelic variant sites for each individual across the 26 populations in the study. To investigate the impact of admixture, we also calculated the African ancestry proportion for each individual in the six admixed populations (ACB, ASW, CLM, MXL, PEL, and PUR). The proportion of African ancestry was calculated by summing the lengths of all African ancestry tracts for each individual from the analysis by Martin et al. (2017). If there was an African tract on both chromosomes, then the total length of this tract was used, whereas if there was an African tract on only one chromosome, then half of the tract's length was used. Lastly, we divided the length of all African ancestry tracts by the total length of all ancestry tracts to account for the small percentage of tracts that had unknown ancestry. For the rest of our analyses, we considered ACB and ASW as African populations due to their high proportion of African ancestry, and so we will use the term “admixed American populations” to refer to the other four admixed populations in the 1000 Genomes data set (CLM, MXL, PEL, and PUR).

We also sought to uncover levels of heterozygosity and counted the number of biallelic heterozygous sites for each individual across the autosomes and the X chromosome. When counting on the X chromosome, we only considered positions within the pseudoautosomal regions, where males and females are both diploid. To examine the distribution of heterozygous sites within admixed genomes, we divided the genome of the individuals from admixed American populations into regions for each combination of ancestry calls (both chromosomes African, one African and one European chromosome, one African and one Indigenous American chromosome, both chromosomes European, one European and one Indigenous American chromosome, and both chromosomes Indigenous American), according to the ancestry tracts determined by Martin et al. (2017). The proportion of heterozygotes in each ancestry tract for each individual was calculated and then averaged across all tracts of a given ancestry type for each individual. A Dunn test was used to determine if the distribution of average heterozygote proportions differed for each ancestry type, and a Bonferroni correction was used.

### Characterizing Archaic Introgression in 1000 Genomes Populations

To quantify the amount of archaic ancestry present in each individual and population, we used two methods that employed the counting of alleles that are shared with archaic humans. First, we used the list of archaic sites identified by Browning et al. (2018a), filtered out all sites that were not biallelic, and counted all positions that contained an archaic allele (any position with a “match” to Neanderthal or a Denisovan), as well as Neanderthal-specific (a “match” to Neanderthal and a “mismatch” to Denisovan) and Denisovan-specific sites (a “mismatch” to Neanderthal and a “match” to Denisovan). Second, we compared the 1000 Genomes population data with the Denisovan and Altai, Chagyrskaya, and Vindija Neanderthal genomes. The archaic SNP calls were filtered for a minimum genotype score of 40. An allele was then considered “archaic” if it was shared with an archaic individual (Denisovan-specific, Neanderthal-specific, and all archaic alleles were considered) and found at a low frequency in Africa (<0.01), but had a frequency of 0.01 or greater in at least one non-African population (Witt et al. 2022). We will refer to this method of archaic allele counting as the “allele frequency counting method.” For both methods, allele counts were made per individual per allele (how many archaic alleles were present), as well as per position (how many positions contained archaic alleles).

### Assessing Impact of Ancestry on Archaic Allele Distribution

To assess whether admixed ancestry impacts the amount of archaic ancestry in an individual, we used the ancestry designations discussed previously (from Martin et al. 2017) and separated the tracts based on the diploid ancestry call (e.g., two chromosomes with African ancestry, one chromosome with African ancestry, and one chromosome with European ancestry). We counted the number of positions containing archaic alleles using sPrime (Browning et al. 2018a, 2018b), including all archaic alleles, Neanderthal-specific alleles, and Denisovan-specific alleles in each type of ancestry tract, and calculated the allele density for each ancestry tract in each individual by dividing the archaic position count by the tract length. To calculate the average archaic density for each ancestry type in each individual, we used the following equation:


$$\frac{\mathrm{Number}\;\mathrm{of}\;\mathrm{SNPs}\;\mathrm{with}\;\mathrm{archaic}\;\mathrm{alleles}\;\mathrm{in}\;\mathrm{tracts}\;\mathrm{with}\;X\;\mathrm{ancestry}}{\mathrm{Total}\;\mathrm{length}\;\mathrm{of}\;\mathrm{tracts}\;\mathrm{with}\;X\;\mathrm{ancestry}}.$$

We also compared archaic allele density of individual tracts by examining the ancestry tracts with the top 1% for archaic, Neanderthal, and Denisovan allele density. For this analysis, we focused specifically on regions where both chromosomes had the same ancestry (African, European, or Indigenous American). One tract with African ancestry was in the top 1% for Denisovan allele density (chromosome 10, 10:13980030-15983537, with nine Denisovan alleles between 14080000 and 14090000), and we further examined that segment to determine if the ancestry was miscalled or if it was ancestral standing variation. We used haplostrips (Marnetto and Huerta-Sánchez 2017) to compare the Denisovan haplotype with that of American and African populations and looked specifically at the individuals with 15 or fewer differences from the Denisovan haplotype (supplementary fig. S6, Supplementary Material online). Another tract with European ancestry had a high density of Denisovan alleles in a single individual at chromosome 6:30800326-33628922, and we examined the haplotypes in admixed American populations, GRB, YRI, and CHB at that locus (supplementary fig. S7, Supplementary Material online).

We determined if Indigenous American ancestry tracts with high archaic allele density were shared between individuals by taking the ancestry tracts with the top 1% allele densities and examining them for overlap of at least 50 kB. If multiple 50-kB tracts all had high allele density, we merged them into one larger tract. The overlapping tracts with the top 5% of greatest sharing between individuals are reported here. This analysis was repeated for all archaic variants as well as Denisovan- and Neanderthal-specific variants—the analysis examining all archaic variants returned the same results as the one analyzing Neanderthal variants, so these regions are reported as being specifically Neanderthal in origin.

To find candidate genes for archaic introgression in each admixed American population, we identified alleles using the “allele frequency counting method” and calculated the PBS between admixed American populations, CHB (the comparison population), and CEU (the outgroup population). We calculated PBS only for sites that had an archaic allele frequency greater than 20% in the admixed population. To calculate *F*_ST_, we used the Weir and Cockerham estimator (from 1984) implemented by vcftools. PBS for each archaic site was calculated according to the following formula:


$$\frac{T_{\mathrm{AMR}/\mathrm{CHB}\;}\;+\;T_{\mathrm{AMR}/\mathrm{CEU}\;}\;-\;T_{\mathrm{CHB}/\mathrm{CEU}\;}}{2},$$

where $T\;=\;-\log(1\;-F_{\mathrm{ST}\;})$ (Yi et al. 2010).

To determine the threshold for outlier archaic sites, we calculated PBS for 2 million random sites across the genome (without replacement) and set an initial threshold at the cutoff for the top 1% of PBS values for the random sites.

We also checked which genes would have significant PBS values when we used Siberians as the comparison population instead of CHB. For this analysis, we used genomes from the Aleut, Eskimo_Chaplin, Eskimo_Naukan, Eskimo_Sireniki, Mansi, and Tlingit populations from the Simons Genome Diversity Project (Mallick et al. 2016), for a total of ten individuals. These populations are all either Siberian or located in close proximity to the Siberian populations. We randomly sampled ten individuals from MXL and ten from CEU to use as a size-matched comparison, then calculated PBS statistics using the above methods. Each allele had to have a minimum frequency of 5% in the Mexican sample, and there had to be at least ten SNPs with a significant PBS value (above 0.09, the curoff established from simulating neutral mutations) within a gene for a gene to be considered as having a significant allele frequency difference between Mexicans and Siberians.

### Verifying PBS Value Significance Using Forward-in-Time Simulations

To assess whether our results could also reflect alleles that are neutral and have risen in frequency due to demographic effects, we used neutral simulations to determine an appropriate threshold cutoff. We focused on the Mexican population because parameters have previously been inferred for admixture in that population (Browning et al. 2018b) and simulated genomic regions that mimic the longest and shortest gene we identified through the initial PBS analysis: a region including *MUC19* (750,000 bp length, chr12:40,269,000–41,019,000) and a region including *VNN1* (145,000 bp, chr6:132,911,000–133,056,000). We used the software SLiM (version 3.7.1) (Haller and Messer 2019) to mimic these tracts with the genetic structure obtained from the modern human genome (GRCh37/hg19 build). Both genomic regions were divided into exons and blocks corresponding to introns or other noncoding regions of the genome. We used the exon ranges defined by the GENCODE v.14 annotations (Harrow et al. 2012) and defined regions between exons as neutral blocks. We conducted neutral mutations by introducing a neutral mutation type with selection coefficient, *s* = 0, at a 1:1 ratio on exons and neutral/noncoding blocks.

Because a demographic model that accurately explains the complex demographic history of the admixed American populations has not been inferred, we expanded the demographic model inferred by Gutenkunst et al. (2009) to model the demographic history of the Mexican population. We included parameters from Jacobs et al. (2019) and Malaspinas et al. (2016) to account for archaic (Altai Neanderthal and Altai Denisovan) introgression. We also incorporated parameters proposed by Browning et al. (2018b) simulations to include European and African admixture into MXL owing to colonization. Therefore, our model integrates Africa/Europe/Asia and Asia/Mexico peopling, including archaic introgression and European colonization (supplementary table S3 and fig. S10, Supplementary Material online).

The model consists of an archaic population that splits early in human history from an ancestral/root population in equilibrium (pre-Africa). After a period of time (5,135 generations), the archaic population splits into two subpopulations (DEN for Altai Denisova and NEN for Altai Neanderthal). Subsequently, the ancestral/root population (AFR) has a sudden increase in population size. Three thousand and two hundred generations after the African expansion, one subpopulation (OoA for Out of Africa Bottleneck) splits from AFR and experiences a reduction in size. A 10% pulse of admixture (lasting one generation) occurs from NEN to OoA and some time after, the OoA population splits again into Europe (EUR) and East Asia (EAS). Right after the EUR–EAS split, another 10% pulse of admixture (lasting one generation) occurs from DEN to EAS. Finally, the EAS subpopulation splits again into EAS and Mexico (MXL) and 12 generations before the present, a single pulse of admixture occurs from EUR to MXL and another one from AFR to MXL with an admixture proportion of 16% and 33%. Note that this demographic model was not inferred, and the mutation rate that we are using is from Gutenkunst et al. (2009) (μ = 2.35e−08). We ran the simulations using two types of recombination rates: the sex-averaged recombination map defined by Kong et al. (2010) averaged over a 10-kb scale and a uniform recombination rate averaged at 1.95412e−08 for the shortest tract and 3.98707e−09 for the longest tract.

For comparison purposes, we obtained 20 simulation replicates for each case. We then calculated the PBS score (Yi et al. 2010) for each SNP position. We used MXL as the population of interest, EAS as the closely related population for comparison, and EUR as the outgroup population. We used the scikit-allel package (Miles et al. 2020) to calculate FST values.

### Comparing Archaic Ancestry Tracts with Modern Ancestry Tracts

To provide additional support to our analysis of archaic SNPs, some of which may be the product of incomplete lineage sorting, we confirmed some of our analyses using archaic ancestry tracts. We used two sets of archaic introgression tracts for our comparison: the first set was created using the hidden Markov model-based method developed by Skov et al. (2018), using a likelihood cutoff of 0.9, and the second set was created using a conditional random field-based method by Sankararaman et al. (2014). The introgression maps from Sankararaman et al. were made for PUR, CLM, and MXL but not PEL. To confirm our analysis of archaic ancestry density, we compared the overlap between modern ancestry regions and archaic introgression tracts. First, we divided the genomes of the admixed American individuals into diploid ancestry regions (i.e., both chromosomes in the region had African ancestry, one chromosome in the region had European, and one chromosome in the region had African ancestry). For each ancestry combination, we summed the total diploid ancestry length and the total length of overlap with an archaic introgression tract for each individual and calculated the % overlap with archaic ancestry for each modern ancestry combination for each individual. We also examined whether Denisovan ancestry was found at higher frequencies in regions of Indigenous American ancestry by focusing on introgressed regions from Skov et al. (2018) that shared more SNPs with Denisovans than with Neanderthals and calculating the percentage of an archaic introgressed tract that was contained within a modern ancestry tract of a given type. We focused on homozygous ancestry tracts because the archaic introgression tracts were diploid.

We also assessed how often an archaic SNP with high PBS values overlapped with an identified archaic ancestry tract. We focused on MXL and PEL for this analysis, as they had more significant PBS results, and calculated how often an individual with the archaic allele at a SNP also had an archaic ancestry tract identified in the region. We used Skov et al. (2018) for our archaic introgression map for this analysis.

### Identifying Regions of Local Adaptation with Archaic Alleles

To investigate sites that might contribute to adaptive archaic introgression of genes, we first identified sites with archaic alleles using the “allele frequency counting method” noted above. We used this method to expand the set of SNPs identified as archaic using sPrime, as other studies have indicated that the sPrime algorithm misses some alleles that have previously been identified as archaic in origin (Browning et al. 2018a; Zhang et al. 2021). Once those alleles were identified, we calculated PBS for each archaic site using PEL or MXL as the AMR population, CHB as the comparison population, and CEU as the outgroup. We also calculated the allele frequency difference of the AMR population minus CHB for each archaic allele. To calculate a cutoff value for significance, we also calculated PBS and the AMR minus CHB allele frequency difference for all alleles that are rare in Africa but not shared with archaic humans. To determine candidates for local adaptive archaic introgression, we used genes with greater than ten sites above the mean of the nonarchaic alleles plus two standard deviations. We also compared the allele frequency difference between PEL/MXL and CHB for archaic alleles with the allele frequency difference for alleles that were rare in Africa.

### Checking Function and Impact of Archaic Introgressed Alleles

We took the list of all potentially introgressed genes with significant PBS values in MXL and PEL and ran a GO enrichment analysis on the set of genes by comparing it with the PANTHER database (Mi et al. 2013). We also used SnpEff to annotate the archaic SNPs within those genes to assess how many were intronic or exonic, and whether the exonic SNPs altered the amino acid sequence or protein function (Cingolani et al. 2012). In cases where a SNP was annotated with multiple categories, it was classified using the following hierarchy: exon > intron > antisense > protein coding > intergenic > other. A classification of “protein coding” meant that SnpEff classified a SNP as part of a protein-coding region but did not specify whether it was intronic or exonic.

## Supplementary Material

evad066_Supplementary_Data

## Data Availability

The data used for this article include the 1000 Genome Project Phase 3 vcf files (ftp://ftp-trace.ncbi.nih.gov/1000genomes/ftp), the Neanderthal and Denisovan vcf files (http://cdna.eva.mpg.de/neandertal/Vindija/VCF/), ancestry tract data of the admixed American 1000 Genome Project individuals from Martin et al. (2017) (https://personal.broadinstitute.org/armartin/tgp_admixture/), and SNP calls using sPrime from Browning et al. (2018a) (https://data.mendeley.com/datasets/y7hyt83vxr/1). All code used in this project can be found at github.com/SciFunk/ArchaicAdaptationAdmixture and github.com/Kelsey-witt/archaic-modern-admixture.
