# Supplementary material for: The Impact of Modern Admixture on Archaic Human Ancestry in Human Populations
Source: Genome Biol Evol. 2023 Apr 27;15(5):evad066. doi: 10.1093/gbe/evad066 (PMC10194819; doi:10.1093/gbe/evad066)
Supplement: evad066_Supplementary_Data [file evad066_supplementary_data.pdf]

Supplemental Figures

S1: A schematic that illustrates the proportion of African (AFR), European (EUR), and Indigenous American (NAT) ancestry for each of the individuals in the CLM, MXL, PEL, and PUR populations from the 1000 Genomes dataset. Individuals are color-coded by population. Each side of the triangle represents a different type of ancestry, and individual ancestry values are calculated from tracts inferred by Martin et al. (2017)

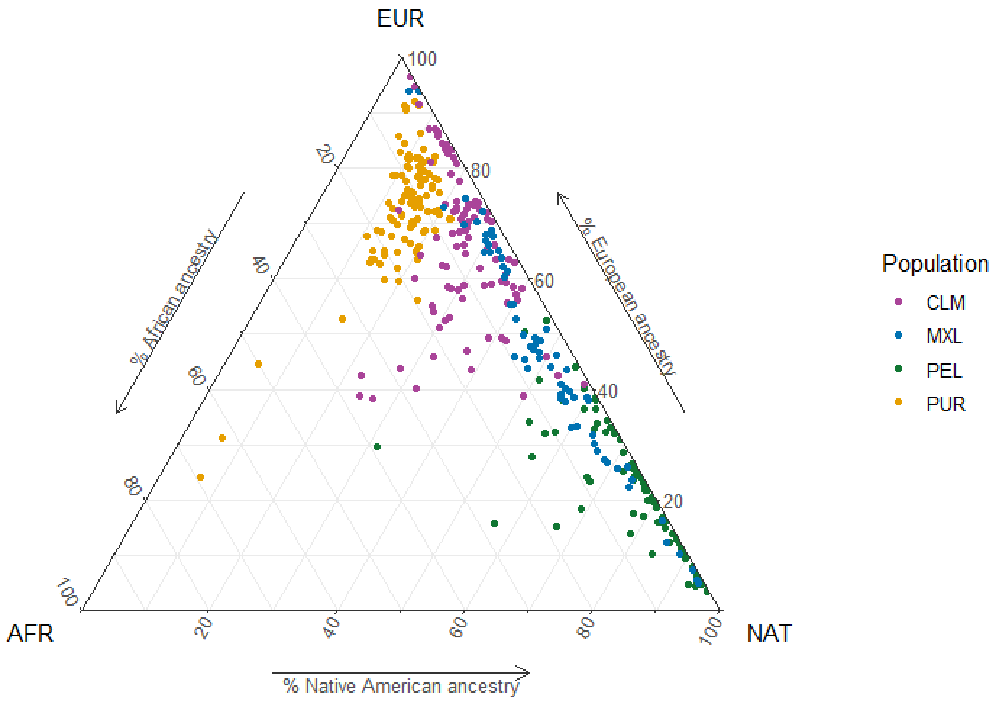

S2: The number of heterozygous sites identified for each of the individuals in the 1000 Genomes Project. The individuals are color-coded by super-population.

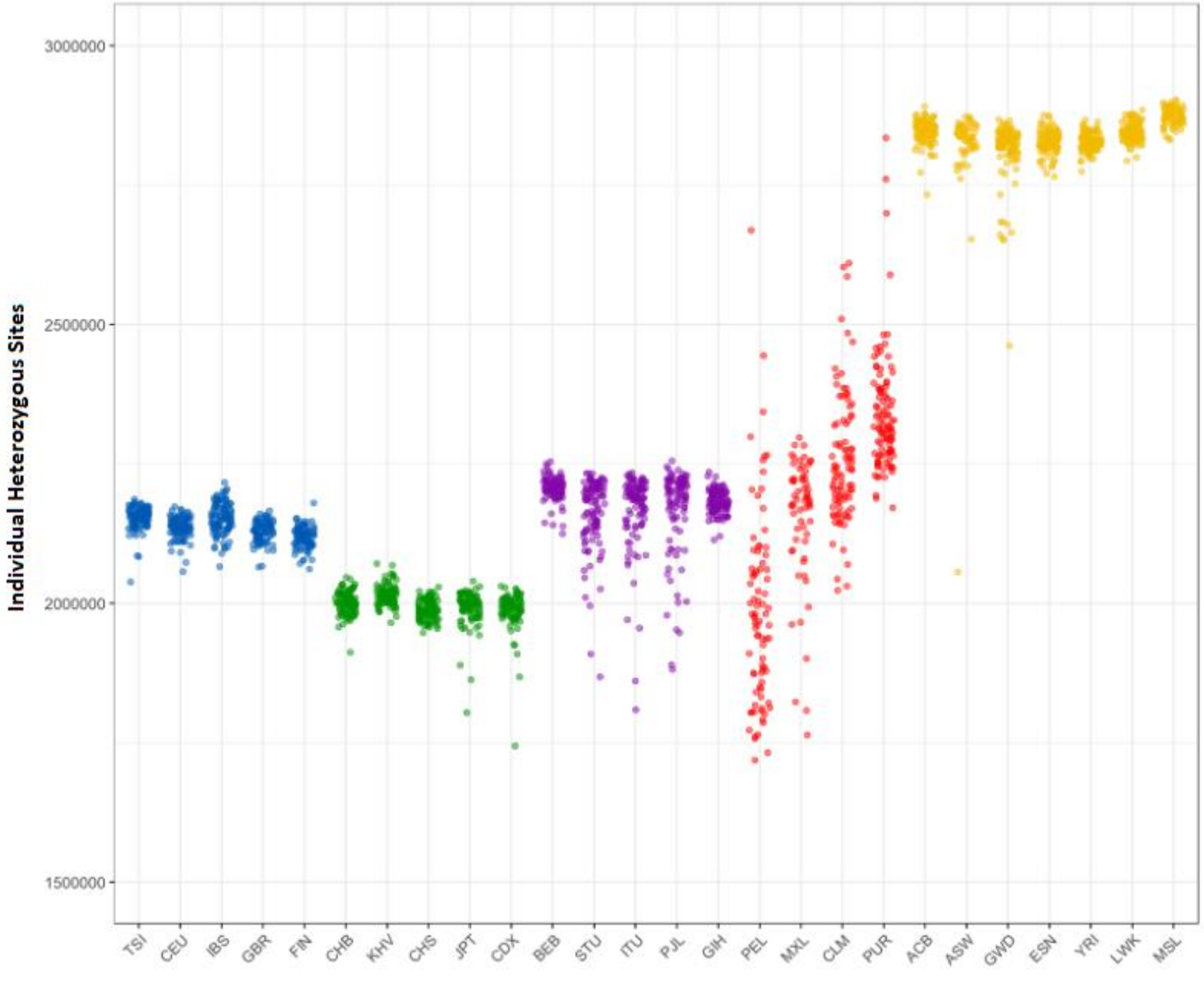

S3: The proportion of heterozygous sites for each diploid ancestry designation for each individual. The average count of heterozygotes was calculated by summing the number of heterozygous sites across every tract with a given ancestry designation, and dividing them by the total length of the ancestry tracts.

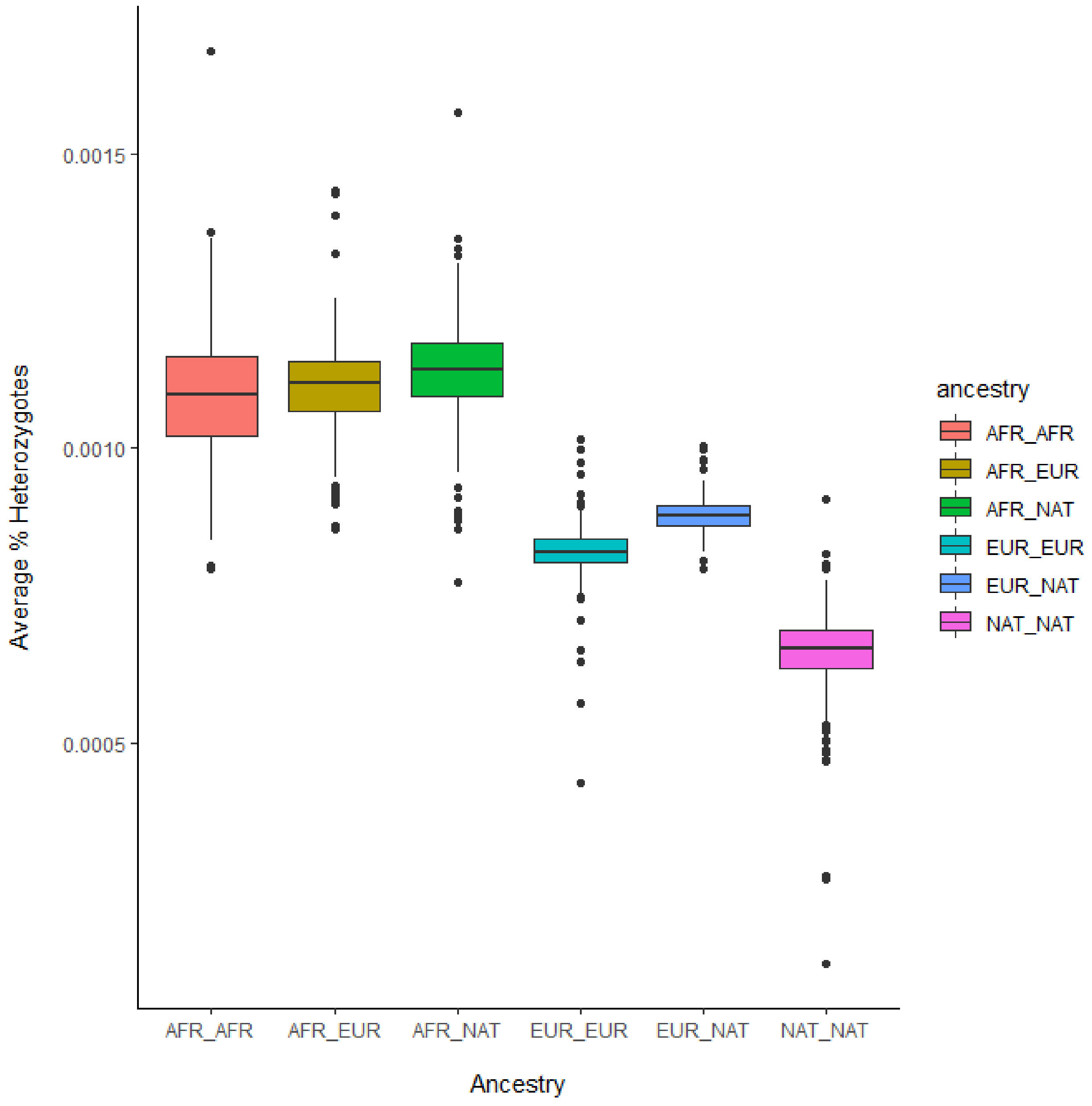

S4:A comparison of the amount of European, Indigenous American and archaic ancestry identified in the admixed American individuals from the 1000 Genomes project dataset (PEL, MXL, CLM, PUR). Each point represents an individual, and the X axis represents the amount of European ancestry, the Y axis represents the amount of Indigenous American ancestry, and the color of the point represents the number of archaic SNPs present in that individual, with darker colors representing more archaic ancestry. Any individuals that fall below the y=1-x line have some amount of African ancestry, which is not depicted here.

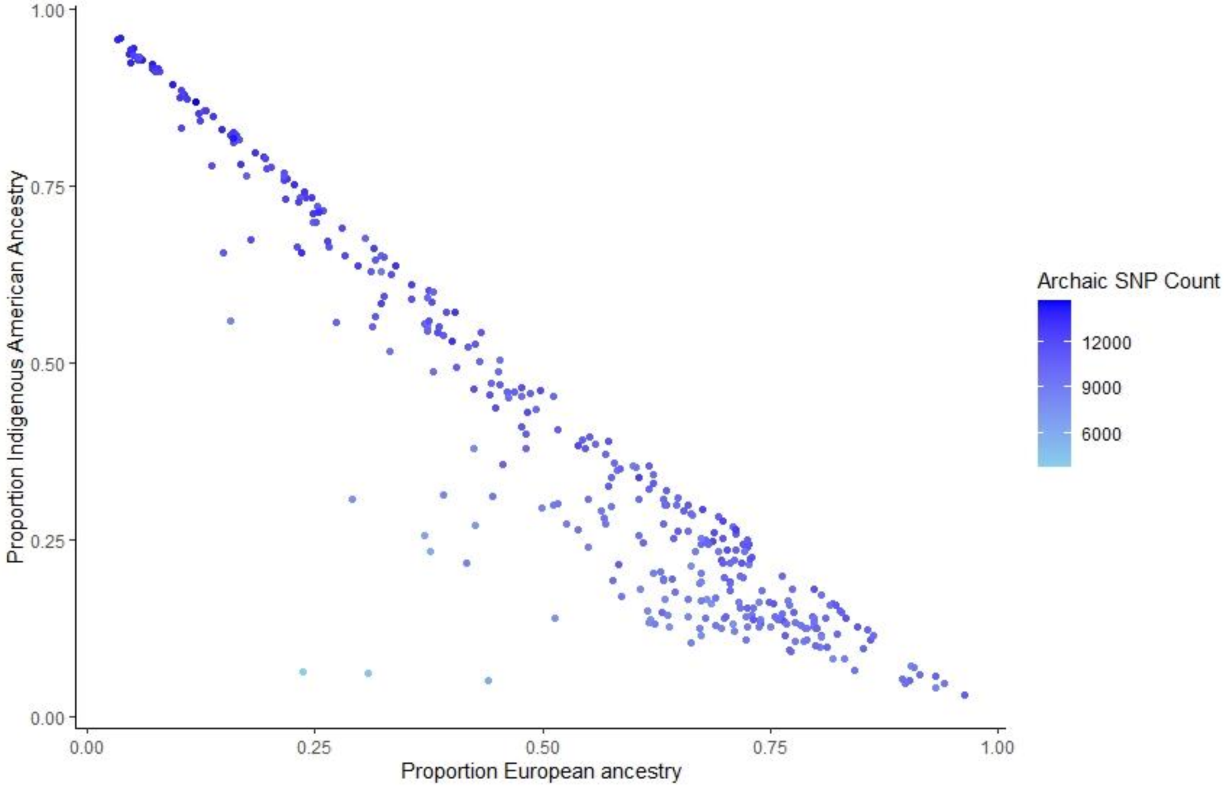

S5: The archaic allele densities of the top 1% of ancestry tracts across all individuals and populations in the Americas. Only regions with homozygous ancestry calls (such as European-European) were considered for this analysis. From top to bottom, the plots reflect Neanderthal-specific allele density, Denisovan-specific allele density, and all archaic allele density. All of the top 1% of tracts had European or Indigenous American ancestry with the exception of one African segment, which had a high proportion of Denisovan-specific ancestry.

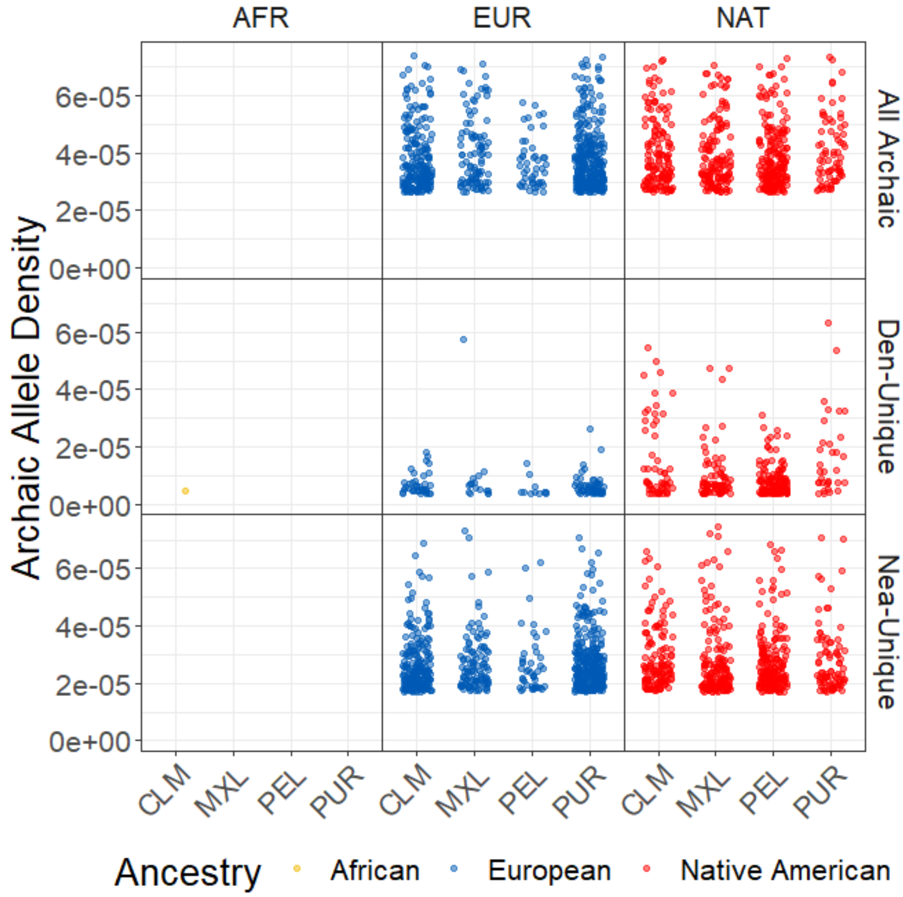

S6: A Haplostrips plot of the African and American haplotypes most similar to the Denisovan haplotype for the African ancestry tract that was in the top 1% for Denisovan allele density (see Fig S4). A) A visual representation of the haplotypes, with black representing an alternative allele and white representing the reference allele. Each horizontal line represents a haplotype, and is color-coded by population - the haplotypes are ordered in increasing distance from the Denisovan haplotypes. The Colombian haplotype specifically is shown in green, and seems to be most similar to other African haplotypes and not the Denisovan haplotype. B) A plot illustrating the distance of each of the haplotypes from the Denisovan reference.

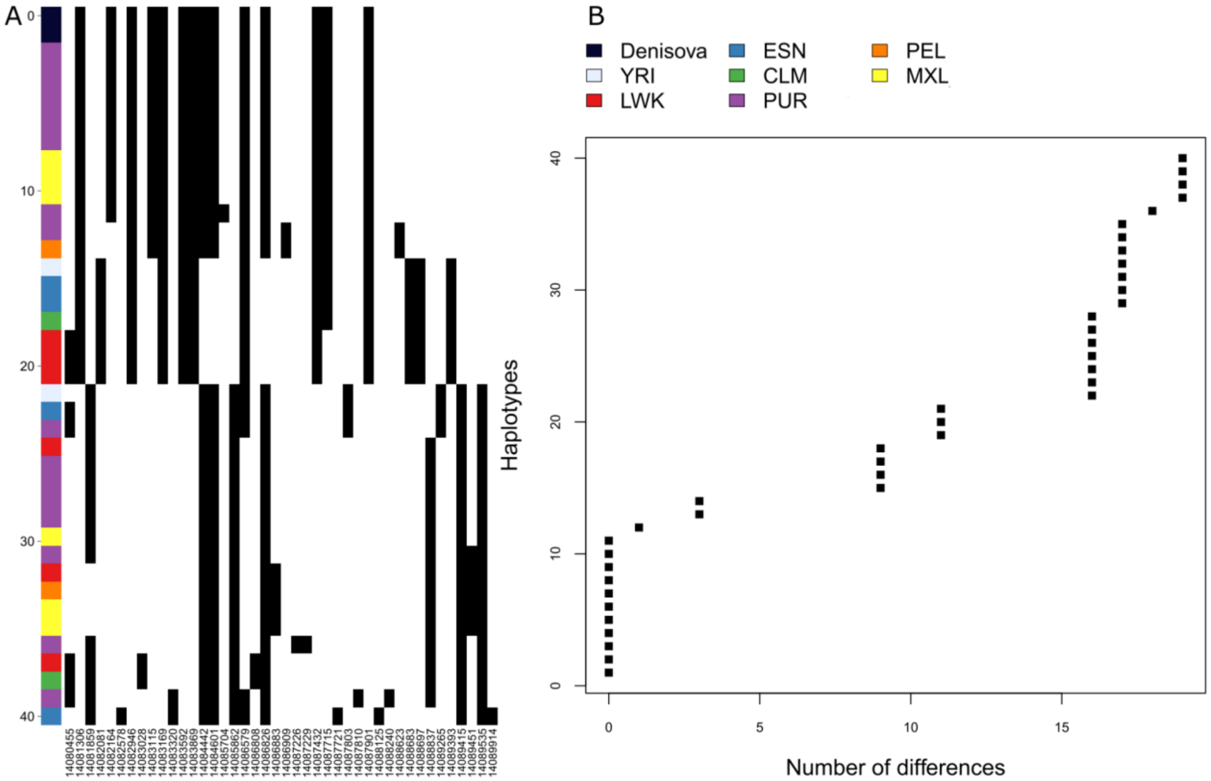

S7:A Haplostrips plot of the region with high Denisovan density in a European ancestry tract (6:30800326-33628922) (see Fig S4). The haplotypes are color-coded, with black representing a derived allele and white representing the ancestral allele. Each horizontal line represents a haplotype, and is color-coded by population - the haplotypes are ordered in increasing distance from the Denisovan haplotypes.

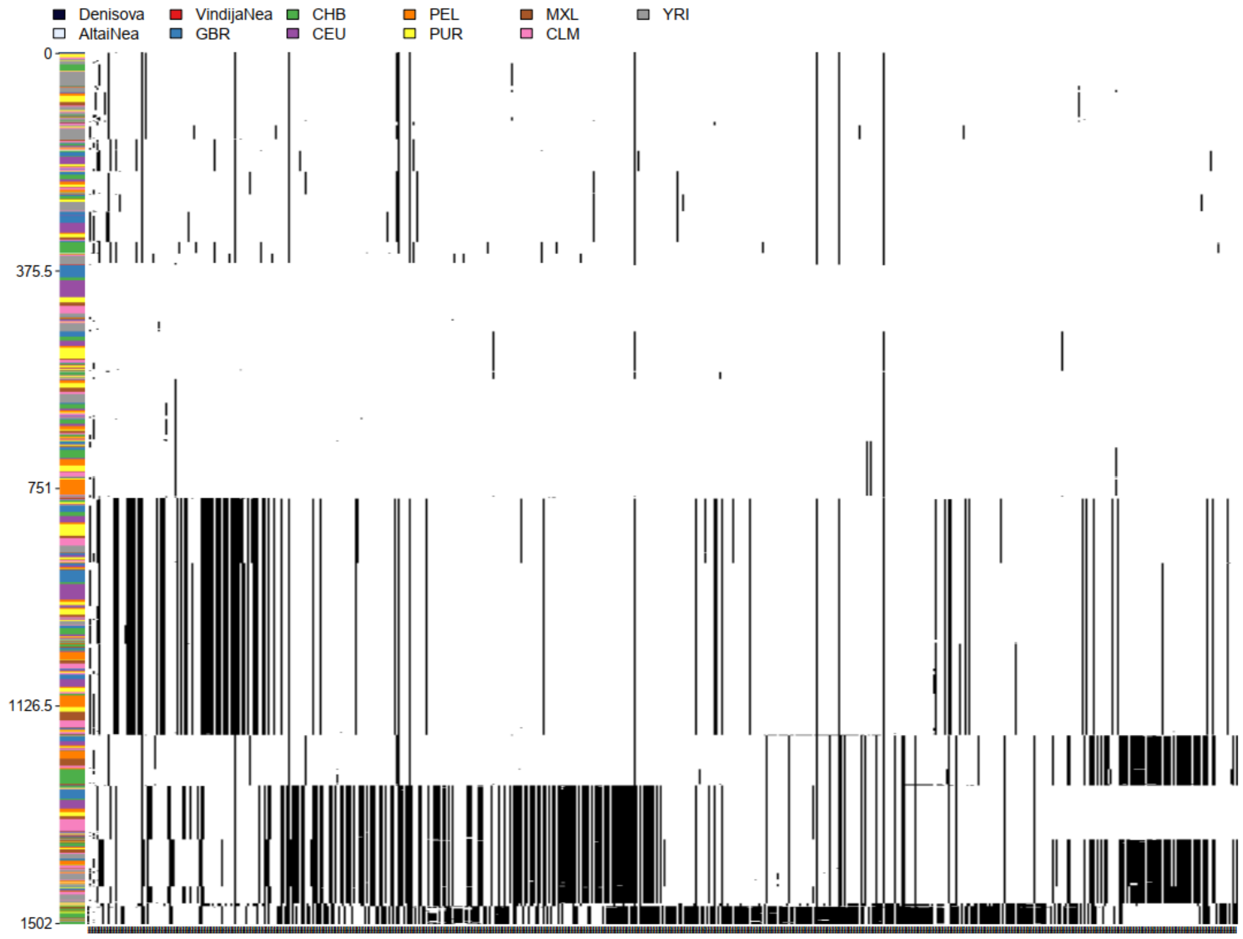

S8: A pie chart that illustrates how the 2,164 SNPs in the genes with significant PBS values are distributed across intergenic, intronic, exonic, and other regions of the genome. Annotations were made using SnpEff (Cingolani et al. 2012). Here “Protein Coding” indicates that SnpEff annotated the SNP as within a coding region but did not specify whether they were intronic or exonic.

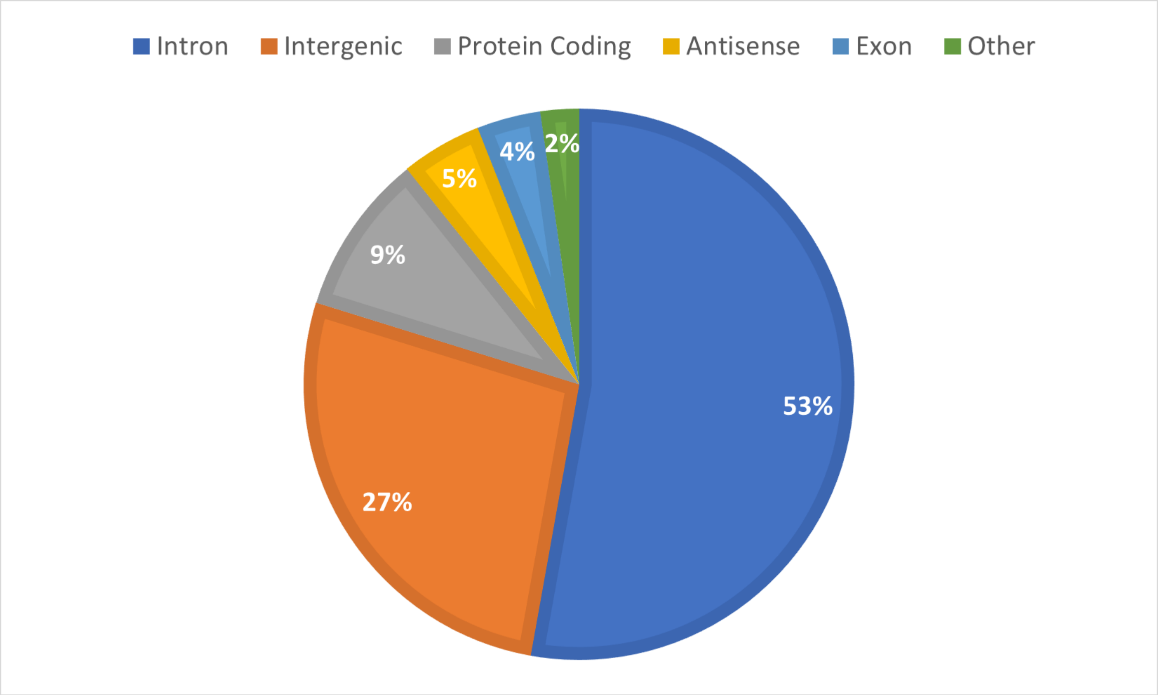

S9: Density plots. Non-archaic rare in Africa sites in grey, archaic rare in Africa sites in purple. The top plot shows the difference in allele frequency between MXL and CHB, the bottom plot is the allele frequency difference between PEL and CHB. Both plots show only SNPs with an archaic allele frequency greater than 5%. The blue line is the value of the random sites mean plus two standard deviations.

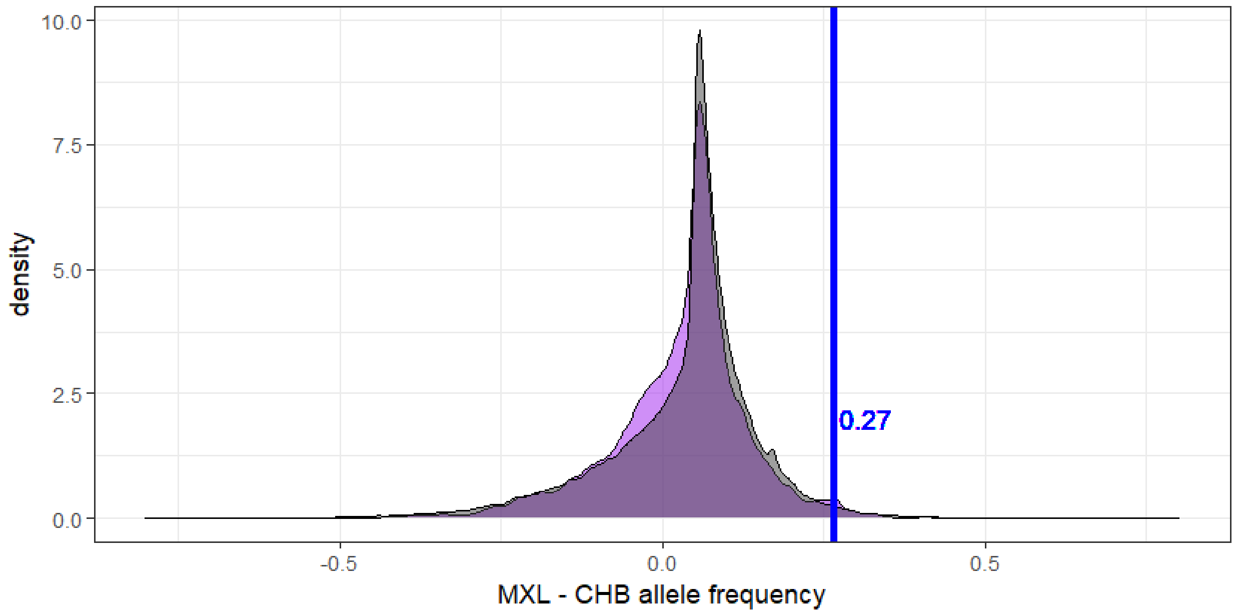

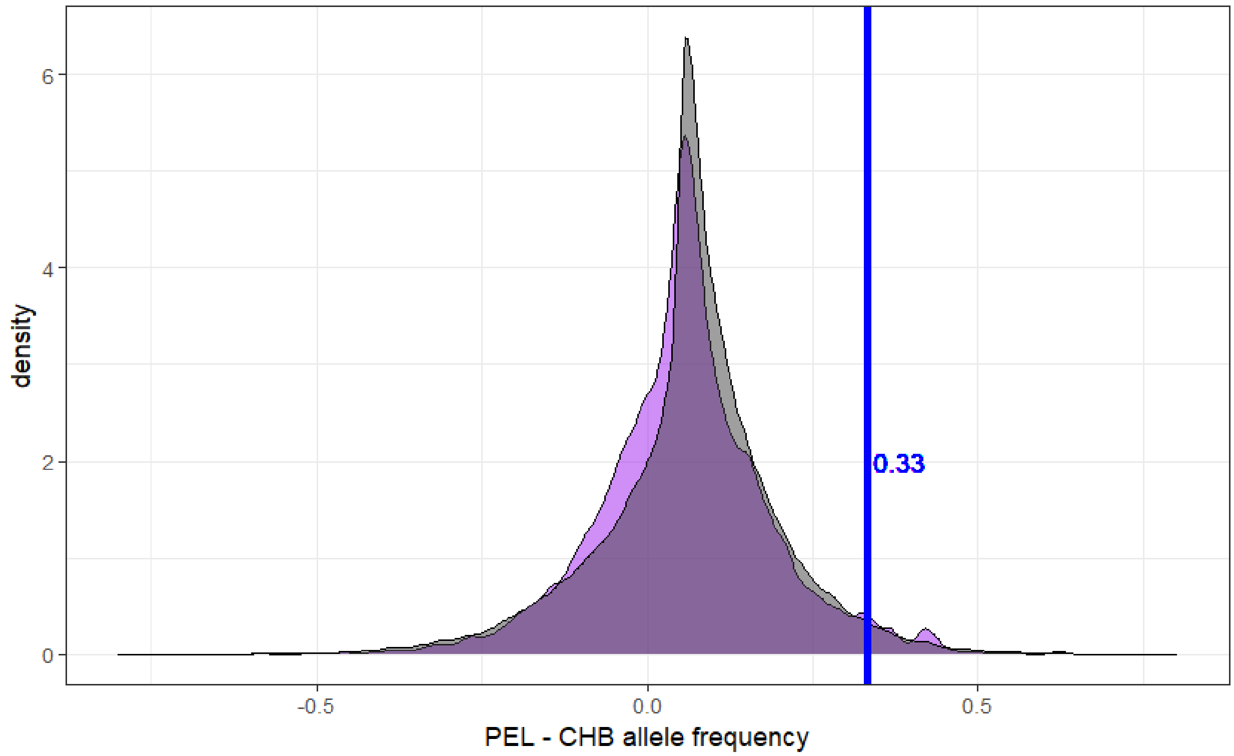

S10: PBS results comparing MXL with Siberians (with CEU as outgroup). Labeled genes have at least 10 alleles with PBS values above the threshold, and were also identified in the PBS analysis with CHB as the comparison population. The horizontal line shows the threshold derived from the top 5% of PBS values from the neutral simulation results. Grey and purple alternate to show chromosomes.

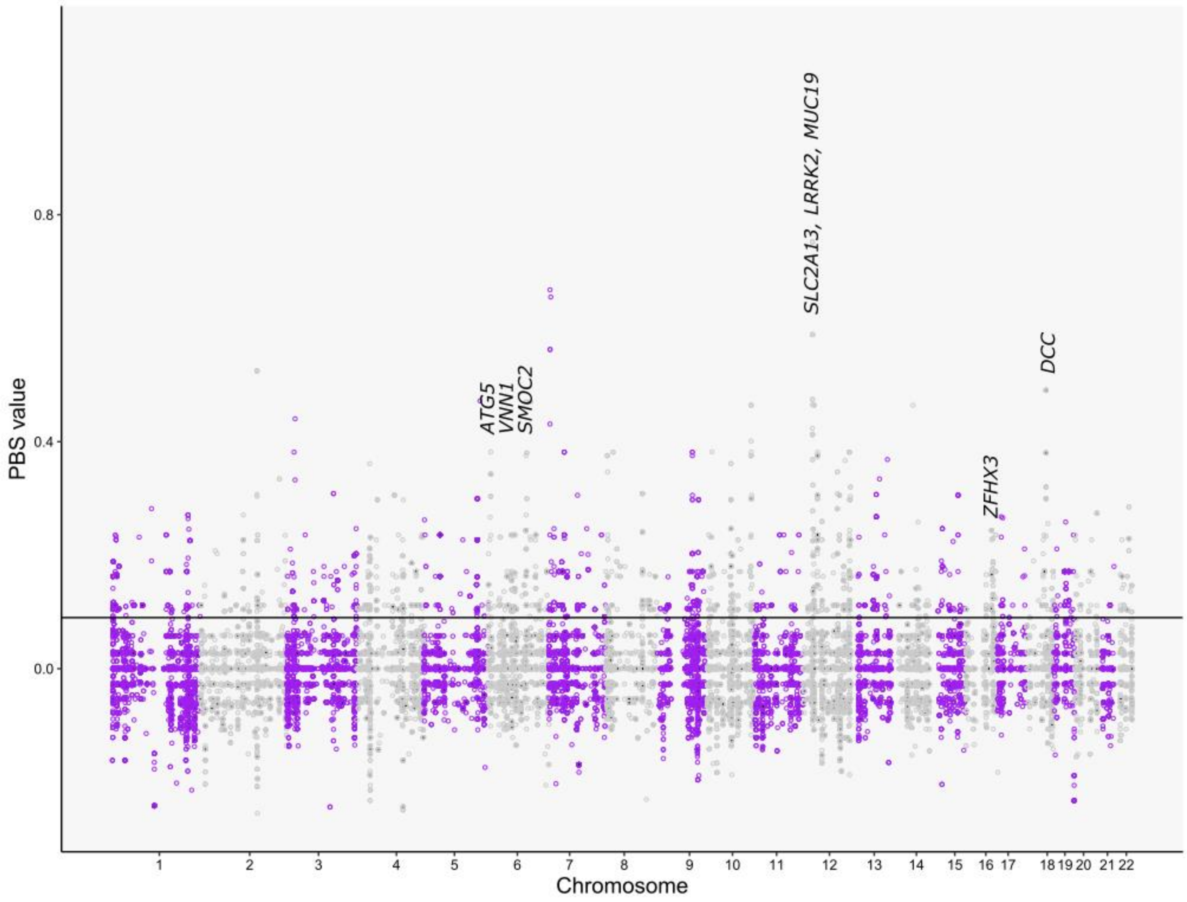

S11: Demographic model for MXL, taken from Gutenkunst et al. 2009. This model extends the Gutenkunst et al. model of African/European/Asian/MXL demographic history to include archaic introgression and admixture owing to European colonization. A complete description of the model can be found in Methods. The AMR (MXL) population has an initial size of 800 and grows at a rate of 0.5% per generation. 12 generations before the present, AMR has a pulse of admixture of ⅙ of the population of African ancestry and ⅓ European. Black arrows represent a single pulse of admixture from X to Y. Gray arrows represent continuous migration from X to Y. Migration rates are represented by m. Note that this demographic model was not inferred. Supplementary Table S2 lists all the parameters of the demographic model.

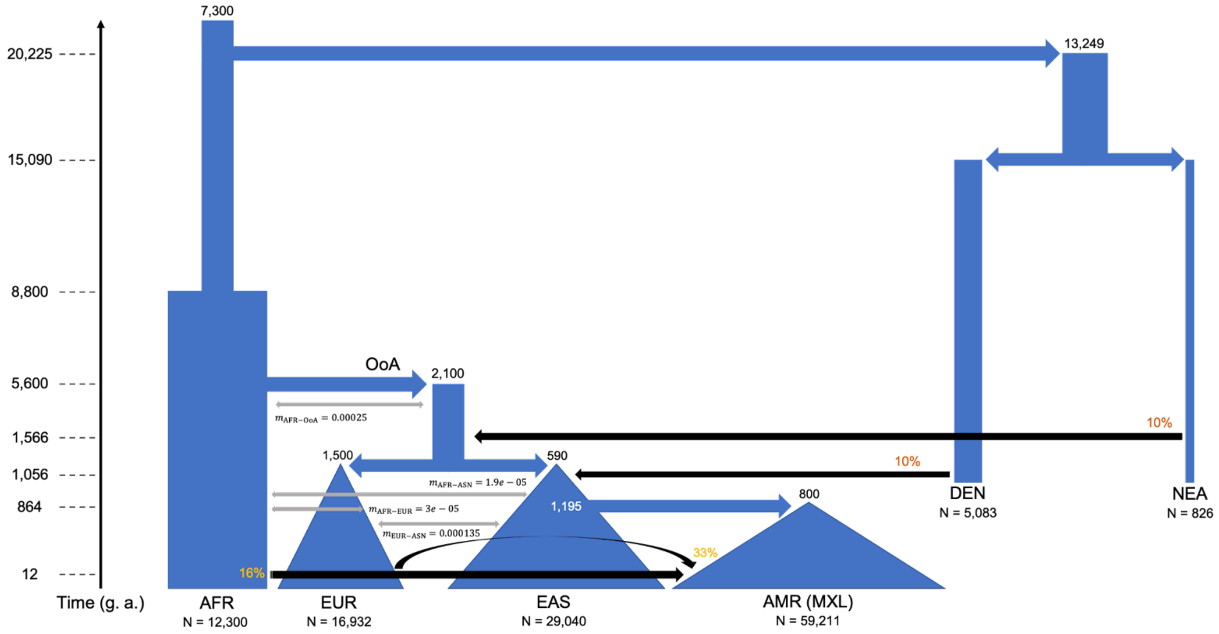

S12 Fig: PBS Simulation Results. We conducted 20 simulations for each scenario and computed the Population Branch Statistic (PBS) score per SNP position comparing the Admixed American population (MXL) with East Asians (Europeans as outgroup). Neutral mutations ($s = 0$) are represented as gray points. The dashed red line stands for the 99th percentile. A) PBS results for the shortest tract, sex-averaged recombination map defined by Kong et al. 2010 averaged over a 10-kb scale (left) and a uniform recombination rate (right). B) PBS results for the longest tract, sex-averaged recombination map defined by Kong et al. 2010 averaged over a 10-kb scale (left) and a uniform recombination rate (right).

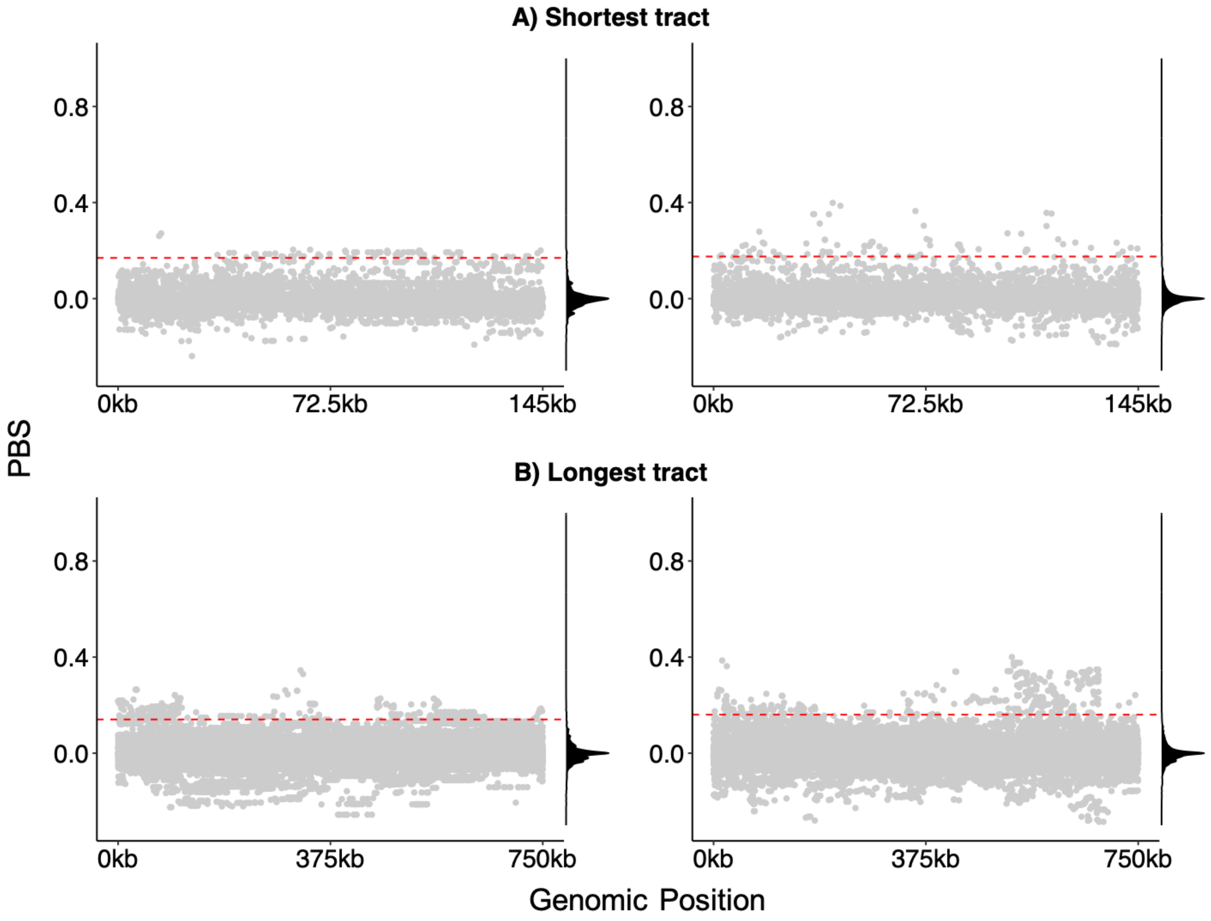

S13 Fig. Archaic Tract Overlap with Diploid Ancestry Tracts. The x axis represents each diploid ancestry type for each population, and the y axis is the percentage of a diploid ancestry tract that overlaps with an archaic ancestry tract identified by A) Skov et al. (2018) using a Hidden Markov Model-based method or B) Sankararaman et al. (2014) using a Conditional Random Field-based method. The total length of all diploid ancestry tracts were summed for each individual. To limit bias due to short ancestry tract lengths, an individual had to have a summed ancestry tract length of at least 100 million base pairs to be included. In Figure B, PEL is excluded because introgression maps were not created for that population.

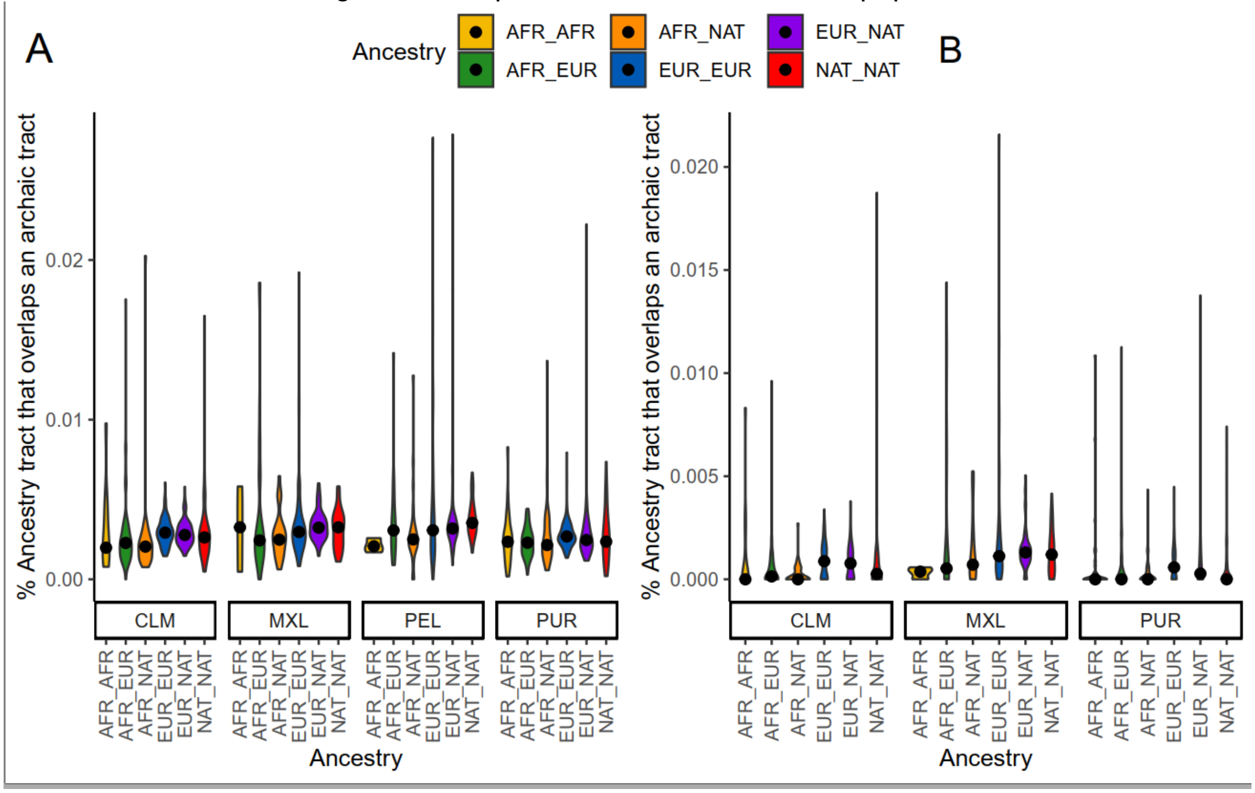

S14 Fig: Denisovan tract overlap with modern ancestry tracts. This figure shows the overlap between each modern ancestry tract and archaic ancestry tract, as identified by Skov et al. (2018). The tracts used here all had more SNPs shared with Denisovans than Neanderthals. A “0” means that the modern ancestry tract did not overlap with a Denisovan ancestry tract, and the other values represent the proportion of a Denisovan ancestry tract that is contained in the modern ancestry tract. Each line shows the density distribution, color-coded by ancestry. The bottom plot is a zoomed-in version of the top plot, to better show the density curves for EUR and NAT.

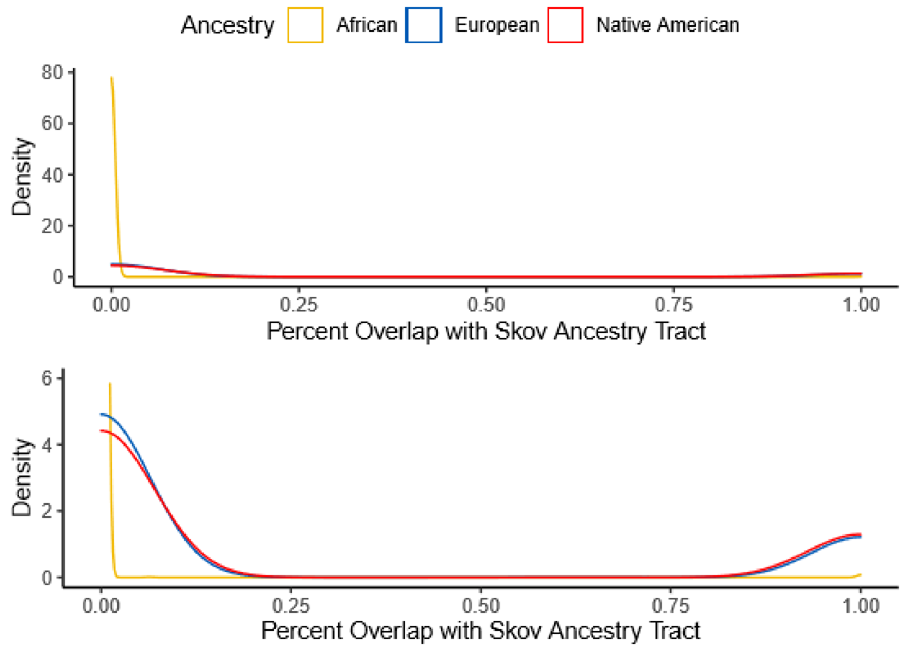

Supplemental Tables

gene	chr	pos	is_it_in_actual_gene	pbs_value
SYT14	1	2.10E+08	no	0.59795
PRDM16	1	3352227	yes	0.455903
FOXO6	1	41786274	no	0.451709
SPRR2F	1	1.53E+08	no	0.355179
TRABD2B	1	48167885	no	0.345883
WARS2	1	1.20E+08	no	0.323295
TNFRSF8	1	12138956	yes	0.308797
KCNH7	2	1.63E+08	no	0.783278
DPP4	2	1.63E+08	no	0.603375
HDLBP	2	2.42E+08	yes	0.598471
SCN9A	2	1.67E+08	yes	0.565145
FAP	2	1.63E+08	yes	0.563915
IFIH1	2	1.63E+08	yes	0.544623
SEPTIN2	2	2.42E+08	yes	0.467383
FARP2	2	2.42E+08	yes	0.456647
EVA1A	2	75659306	no	0.400285
ANO7	2	2.42E+08	yes	0.359927
MYO3B	2	1.72E+08	no	0.348729
PAX3	2	2.23E+08	no	0.343571
ERICH2	2	1.72E+08	yes	0.342955
SP5	2	1.72E+08	no	0.334197
GAD1	2	1.72E+08	yes	0.331402
CXCR4	2	1.37E+08	no	0.313853
SCN1A	2	1.67E+08	no	0.305441
TP63	3	1.90E+08	yes	0.359263
SPSB4	3	1.41E+08	yes	0.33539
ZNF385D	3	21467818	yes	0.323987

KDR	4	55852688	no	0.527888
HAND2	4	1.75E+08	no	0.366549
SPINK1	5	1.47E+08	no	0.523881
GCM1	6	53069840	no	0.417201
FABP7	6	1.23E+08	no	0.405753
PKIB	6	1.23E+08	no	0.405483
ELOVL5	6	53115621	no	0.372273
OSTM1	6	1.08E+08	yes	0.368698
SMPDL3A	6	1.23E+08	yes	0.35151
CRYBG1	6	1.07E+08	no	0.337333
ATG5	6	1.07E+08	yes	0.331702
SMOC2	6	1.69E+08	yes	0.314677
GPR141	7	37626093	no	0.443238
PCLO	7	82307677	no	0.370781
CNTNAP2	7	1.47E+08	yes	0.354371
CLIP2	7	1827427	yes	0.348766
NME8	7	37914716	yes	0.326217
EBF2	8	25666774	no	0.45387
AZIN1	8	1.04E+08	no	0.45092
MATN2	8	98910894	yes	0.444682
ATP6V1C1	8	1.04E+08	no	0.375299
KLF10	8	1.04E+08	no	0.301899
GABBR2	9	1.01E+08	yes	0.363838
HSD17B3	9	98973831	no	0.351555
FBP2	9	97322107	yes	0.308489
GOLGA1	9	1.28E+08	yes	0.308206
SCAI	9	1.28E+08	yes	0.308206
RTKN2	10	63858726	no	0.586684
ADO	10	64598118	no	0.521836

MYPN	10	69926319	yes	0.362178
COL13A1	10	71729613	no	0.33777
DNA2	10	70174885	yes	0.32916
PBLD	10	70044616	yes	0.305431
RUFY2	10	70041263	no	0.305431
SGCG	13	23657942	no	0.624335
FLT1	13	28896979	yes	0.433036
MYO16	13	1.10E+08	yes	0.43068
FREM2	13	39403675	yes	0.410928
KLF12	13	74438244	yes	0.318073
SERPINA6	14	94861399	no	0.352056
ZNF280D	15	56868915	no	0.595203
TCF12	15	57136959	no	0.563547
CGNL1	15	57588129	no	0.542309
UNC13C	15	54702274	yes	0.374606
CHD2	15	93564023	yes	0.323816
ZFHX3	16	72720401	no	0.411188
SLC16A11	17	6915433	no	0.347195
MYOCD	17	12592227	yes	0.339897
CYP2B6	19	41614028	no	0.376871
ZNF787	19	56595551	no	0.344506
CAMSAP3	19	7669229	yes	0.316619
TIAM1	21	32406799	no	0.475382
KRTAP19-8	21	32357175	no	0.46047
DNAL4	22	39287355	no	0.533014
NPTXR	22	39290495	no	0.533014
ZC3H7B	22	41798520	no	0.47548

Table S1: A table listing all of the SNPs with a PBS value in the top 1% of genome-wide PBS values between MXL and CHB or PEL and CHB. The closest gene is listed, along with the chromosome and position, as well as the PBS value. It is also noted whether the SNP is within

the gene itself, or simply within 50 kB of that gene

Chromosome	Region Start	Region End	Gene ID	Number of SNPs
1	3234088	3259500	PRDM16	22
1	12138956	12157321	TNFRSF8	15
1	86101484	86300609	ZNHIT6	29
1	210532056	210579523	HHAT	17
3	21467818	21503536	ZNF385D	11
3	143251224	143351493	SLC9A9	19
3	194990363	195188027	ACAP2	182
4	154510903	154543071	TMEM131L	10
6	65254384	66305898	EYS	203
6	74377732	74579124	CD109	13
6	106669139	106801762	ATG5	14
6	133011340	133049865	VNN1	13
6	133731991	133826073	EYA4	22
6	169013720	169036658	SMOC2	18
7	27776727	27846065	TAX1BP1	16
7	37557543	37928948	GPR141	48
7	50790028	50876673	GRB10	20
7	82021134	82054989	CACNA2D1	12
7	95609144	95629805	DYNC1I1	12
8	102754805	102807539	NCALD	17
8	123893790	123995609	ZHX2	15
9	101338331	101346757	GABBR2	14
9	112076447	112093132	EPB41L4B	11
10	11488873	11655954	USP6NL	11
12	28264703	28737797	CCDC91	164

12	31090690	31144537	TSPAN11	16
12	40285822	40495688	SLC2A13	44
12	40622565	40760764	LRRK2	35
12	40787264	40828306	MUC19	162
12	129566340	129771178	TMEM132D	39
12	131006547	131062468	RIMBP2	10
13	74527758	74558237	KLF12	11
15	56828985	57122399	ZNF280D,	18
16	72320307	72915166	ZFHX3,	38
18	43621995	43732618	PSTPIP2	11
18	49962127	50150981	DCC,	61
18	55112314	55193216	ONECUT2	34
19	29878612	29904799	VSTM2B-DT	38
20	15254848	15293599	MACROD2	13
20	44635416	44680581	SLC12A5	12
22	22094880	22253023	MAPK1	10
22	36954610	37002232	CACNG2	15

Table S2: A summary of the genes identified as significant in the PBS analysis with MXL as the target, Siberians as the comparison, and CEU as the outgroup. Each region had to have at least 10 SNPs above the 5% threshold cutoff from neutral simulations to be considered significant. The table lists the location of the region identified, the start and end of the region (based on the location of significant SNPs), the gene name, and the number of SNPs with significant PBA values in the region.

Parameter Type	Value	Description	Reference
Population size	7,300	Ancestral pop. size	Gutenkunst et al., 2009
Population size	13,249	Archaic pop. size	Jacobs et al., 2019, Malaspinas et al., 2016
Population size	12,300	AFR pop.size	Gutenkunst et al., 2009
Population size	5,083	DEN pop. size	Jacobs et al., 2019, Malaspinas et al., 2016
Population size	826	Neanderthal pop. size	Jacobs et al., 2019, Malaspinas et al., 2016
Population size	2,100	OoA pop.size	Gutenkunst et al., 2009
Population size	1,500	EUR pop. size	Gutenkunst et al., 2009
Population size	590	ASN pop. size	Gutenkunst et al., 2009
Population size	800	MXL pop. size	Gutenkunst et al., 2009
Growth rate per gen	0.23 %	EUR pop. growth rate	Gutenkunst et al., 2009
Growth rate per gen	0.37 %	ASN pop. growth rate	Gutenkunst et al., 2009
Growth rate per gen	0.5 %	MXL pop. growth rate	Gutenkunst et al., 2009
Migration rate per gen	25x10^-5	AFR-OoA migration rate	Browning et al., 2018b, Gravel et al., 2011
Migration rate per gen	3x10^-5	AFR-EUR migration rate	Browning et al., 2018b, Gravel et al., 2011
Migration rate per gen	1.9x10^-5	AFR-ASN migration rate	Browning et al., 2018b, Gravel et al., 2011
Migration rate per gen	13.5x10^-5	EUR-ASN migration rate	Gutenkunst et al., 2009
Admixture percentage	10	DEN admixture into ASN	Reich et al., 2010
Admixture percentage	10	NEA admixture into OoA	Jacobs et al., 2019, Malaspinas et al., 2016
Admixture percentage	16	AFR admixture into MXL	Browning et al., 2018b
Admixture percentage	33	EUR admixture into MXL	Browning et al., 2018b
Epoch time (g. a.)	20,225	HUMAN-ARCHAIC split	Jacobs et al., 2019,

			Malaspinas et al., 2016
Epoch time (g. a.)	15,090	NEA-DEN split	Jacobs et al., 2019, Malaspinas et al., 2016
Epoch time (g. a.)	8,800	AFR expansion	Gutenkunst et al., 2009
Epoch time (g. a.)	5,600	AFR-OoA split	Gutenkunst et al., 2009
Epoch time (g. a.)	1,566	NEA admixture into OoA	Jacobs et al., 2019, Malaspinas et al., 2016
Epoch time (g. a.)	1,056	EUR-ASN split	Gutenkunst et al., 2009
Epoch time (g. a.)	1,056	DEN admixture into ASN	Malaspinas et al., 2016, Jacobs et al., 2019
Epoch time (g. a.)	864	ASN-MXL split	Gutenkunst et al., 2009
Epoch time (g. a.)	12	EUR, AFR admixture into MXL	Browning et al., 2018b
Mutation rate	2.35e^-8	Per-base per-generation mutation rate	Gutenkunst et al., 2009

Table S3: A summary of the parameters used in the forward-in-time SLiM simulations that were used to model admixture in the 1000 Genomes Project MXL population.

Tracts	Skov Median	Sankararaman Median
All Populations		
AFR_AFR	0.00225	0
AFR_EUR	0.00231	0.00018
AFR_NAT	0.00225	0
EUR_EUR	0.00279	0.00082
EUR_NAT	0.00290	0.00084
NAT_NAT	0.00309	0.00044
CLM		
AFR_AFR	0.00198	0
AFR_EUR	0.00227	0.00014
AFR_NAT	0.00204	0
EUR_EUR	0.00292	0.00088
EUR_NAT	0.00278	0.00078
NAT_NAT	0.00262	0.00026
MXL		
AFR_AFR	0.00326	0.00037
AFR_EUR	0.00244	0.00053
AFR_NAT	0.00248	0.00071
EUR_EUR	0.00296	0.00112
EUR_NAT	0.00323	0.00130
NAT_NAT	0.00325	0.00120
PEL		
AFR_AFR	0.00207	N/A

AFR_EUR	0.00306	N/A
AFR_NAT	0.00250	N/A
EUR_EUR	0.00307	N/A
EUR_NAT	0.00318	N/A
NAT_NAT	0.00353	N/A
	PUR	
AFR_AFR	0.00235	0
AFR_EUR	0.00229	0
AFR_NAT	0.00215	0
EUR_EUR	0.00268	0.00058
EUR_NAT	0.00246	0.00028
NAT_NAT	0.00236	0

Table S4: The median value of overlap between an archaic ancestry tract and modern ancestry tracts of a given type. This figure plots the overlap for tracts by Skov et al. (2018) using a Hidden Markov Model-based method and Sankararaman et al. (2014) using a Conditional Random Field-based method. The total length of all diploid ancestry tracts were summed for each individual. To limit bias due to short ancestry tract lengths, an individual had to have a summed ancestry tract length of at least 100 million base pairs to be included. PEL is excluded from the Sankararaman set because introgression maps were not created for that population.
